# NS2B/NS3 mutations enhance the infectivity of genotype I Japanese encephalitis virus in amplifying hosts

**DOI:** 10.1371/journal.ppat.1007992

**Published:** 2019-08-05

**Authors:** Yi-Chin Fan, Jian-Jong Liang, Jo-Mei Chen, Jen-Wei Lin, Yi-Ying Chen, Kuan-Hsuan Su, Chang-Chi Lin, Wu-Chun Tu, Ming-Tang Chiou, Shan-Chia Ou, Gwong-Jen J. Chang, Yi-Ling Lin, Shyan-Song Chiou

**Affiliations:** 1 Graduate Institute of Microbiology and Public Health, National Chung Hsing University, Taichung, Taiwan; 2 Institute of Epidemiology and Preventive Medicine, National Taiwan University, Taipei, Taiwan; 3 Institute of Biomedical Sciences, Academia Sinica, Taipei, Taiwan; 4 Department of Veterinary Medicine, National Pingtung University of Science and Technology, Pingtung, Taiwan; 5 Institute of Preventive Medicine, National Defense Medical Center, Taipei, Taiwan; 6 Department of Entomology, National Chung Hsing University, Taichung, Taiwan; 7 Arboviral Diseases Branch, Centers for Disease Control and Prevention, Fort Collins, Colorado, United States of America; National Institute of Allergy and Infectious Diseases, UNITED STATES

## Abstract

Genotype I (GI) virus has replaced genotype III (GIII) virus as the dominant Japanese encephalitis virus (JEV) in the epidemic area of Asia. The mechanism underlying the genotype replacement remains unclear. Therefore, we focused our current study on investigating the roles of mosquito vector and amplifying host(s) in JEV genotype replacement by comparing the replication ability of GI and GIII viruses. GI and GIII viruses had similar infection rates and replicated to similar viral titers after blood meal feedings in *Culex tritaeniorhynchus*. However, GI virus yielded a higher viral titer in amplifying host-derived cells, especially at an elevated temperature, and produced an earlier and higher viremia in experimentally inoculated pigs, ducklings, and young chickens. Subsequently we identified the amplification advantage of viral genetic determinants from GI viruses by utilizing chimeric and recombinant JEVs (rJEVs). Compared to the recombinant GIII virus (rGIII virus), we observed that both the recombinant GI virus and the chimeric rJEVs encoding GI virus-derived NS1-3 genes supported higher replication ability in amplifying hosts. The replication advantage of the chimeric rJEVs was lost after introduction of a single substitution from a GIII viral mutation (NS2B-L99V, NS3-S78A, or NS3-D177E). In addition, the gain-of-function assay further elucidated that rGIII virus encoding GI virus NS2B-V99L/NS3-A78S/E177E substitutions re-gained the enhanced replication ability. Thus, we conclude that the replication advantage of GI virus in pigs and poultry is the result of three critical NS2B/NS3 substitutions. This may lead to more efficient transmission of GI virus than GIII virus in the amplifying host-mosquito cycle.

## Author summary

Flaviviral vertebrate amplifying host(s), invertebrate vector(s), genetics, and environmental factors shape the viral geographical distribution and epidemic disease pattern. Newly emerging dengue virus genotypes, West Nile virus clades, or Zika virus strains exhibited an enhancement in mosquito vector competence. However, hosts and viral determinants responsible for the occurrence of JEV genotype replacement remains unclear. Here, we demonstrated that emerging GI viruses with enhanced transmission potential in amplifying hosts such as pigs and avian species was encoded by three critical GI-specific mutations in NS2B/NS3 proteins. This discovery provides insight into the viral genetic mechanism underlying the GI virus advantage and adaptation in the pig/avian species-mosquito cycle. Our results also emphasize the importance of monitoring viral evolution in amplifying vertebrate hosts to clarify the role of avian species in local transmission of GI virus in JE endemic and epidemic countries.

## Introduction

Japanese encephalitis virus (JEV), a mosquito-borne flavivirus, has a single-stranded, positive-sense RNA genome encoding three structural proteins (capsid, precursor membrane protein, and envelope protein) and seven non-structural proteins (NS1, NS2A, NS2B, NS3, NS4A, NS4B, and NS5) [1]. JEV was first isolated in 1935. Since then JEV has been classified into five genotypes (GI-GV) with variant geographic distribution in Asia and Australia. Traditionally, GIII virus is the most widely distributed and dominant JEV genotype in JEV endemic/epidemic regions [2]. The ecological factors of GIII endemic or epidemic areas are well characterized, and the virus is maintained primarily in the *Culex tritaeniorhynchus-*amplifying host (pigs and avian species) transmission cycle, and humans and horses are accidental, dead-end hosts [3–8]. An estimated 67,900 JE human cases occur annually with a 20–30% case-fatality rate, and 30–50% of surviving patients suffer neurological sequelae [9].

GI virus emerged in the 1990s and has gradually replaced GIII virus as the most frequently isolated JEV genotype from *Culex tritaeniorhynchus*, stillborn piglets, and JE patients in Japan, Korea, Vietnam, Thailand, Taiwan, China, and India [10–15]. We suggest that GI virus might compete with GIII virus in the same pig-mosquito cycle and exhibit a transmission advantage over areas previously dominated by GIII virus [11, 16]. Emerging GI virus replicates more efficiently in *Aedes albopictus* mosquito-derived cells, birds, and ducklings than GIII virus [17–19]; however, it remains unknown if the replication efficiency of GI vs. GIII virus occurs in pigs and/or *Culex tritaeniorhynchus* mosquitoes, which play critical roles in local transmission of JEV. In addition, the experimental evidence associated with the difference in viral genomic factor(s) does not fully support the occurrence of GIII replacement during the past 20 years [11, 18, 20].

In this study, we investigated the *in vitro* and *in vivo* replication characteristics of emerging GI virus and previously dominate GIII virus in *Culex tritaeniorhynchus* mosquito vector and amplifying hosts (pigs, young chickens, and ducklings). We did not include dead-end hosts in this study because of the remote possibility of the involvement of dead-end hosts in the transmission of JEV. More importantly, we constructed, generated and applied various genotypic chimeras of recombinant JEVs from infectious cDNA clones to determine the viral genetic determinants that enhanced and increased the replication efficiency of GI virus over GIII virus. These genetic determinants would be critically important for selection of viral hosts and genes to monitor GI virus activity and evolution in a natural transmission cycle.

## Results

### Replication ability of GIII and GI viruses in *Culex tritaeniorhynchus*

**Fig 1:**
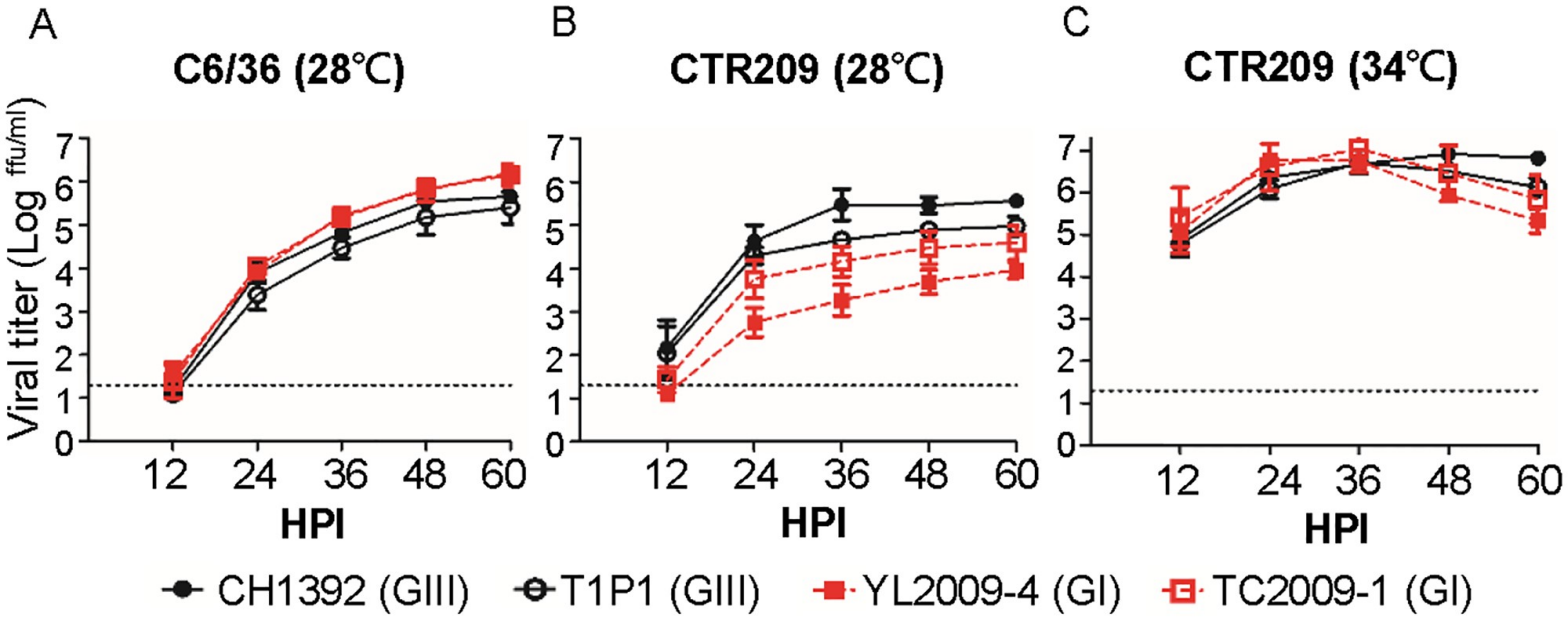
Growth curves of GIII and GI JEVs in *Aedes albopictus* and *Culex tritaeniorhynchus* mosquito-derived cell lines. (A-C) GIII CH1392 virus (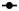), GIII T1P1 virus (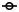), GI YL2009-4 virus (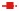), or GI TC2009-1 virus (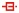)-infected mosquito-derived cells at 0.5 multiplicity of infection (MOI). Virus was amplified in C6/36 cells at 28°C (A) and in CTR209 cells at 28°C (B) or 34°C (C). The viral titer (focus-forming unit [ffu] per ml) was determined in the supernatant at 12, 24, 36, 48, and 60 hours post infection (HPI) by micro-antigen focus assay. Mean, with standard error of the mean (SEM), for triplicates is shown. A dotted line indicates the detection limit. The difference in viral titer was calculated for each time point using one-way ANOVA followed by Turkey’s Multiple Comparison Test.

To analyze the growth curve of GIII and GI JEVs in mosquito-derived cells, we infected *Aedes albopictus*-derived C6/36 cells and *Culex tritaeniorhynchus*-derived CTR209 cells with GIII viruses (CH1392 and T1P1 strains) and GI viruses (YL2009-4 and TC2009-1 strains) at 28°C (Fig 1A and 1B). The growth curves and virus titers from GI and GIII-infected C6/36 cells were similar with no statistical significance between them during a 60-hour infection period (Fig 1A). In CTR209 cells, the growth curves were statistically non-significant differences (p>0.05) between genotypes; although, GI YL2009-4 virus was less efficient and generated an average of 1.61–2.21 log lower viral titer than GIII-CH1392 virus (Fig 1B). In addition, comparable ratios of viral NS3 proteins to α-tubulin were detected in GIII and GI virus-infected C6/36 and CTR209 cells at 48 hours post-infection (HPI) (S1A, S1B, S1D and S1E Fig). The newly evolved WNV genotype (WN02) showed an ability to adapt to mosquitoes reared at a higher temperature [21]. Therefore, we further compared GIII to GI viruses in CTR209 cells at an elevated temperature. When the temperature was increased to 34°C, similar titers for both GIII and GI viruses were observed even though higher relative intensity of NS3 proteins from GI viruses was detected in CTR209 cells (Fig 1C, S1C and S1F Fig). This suggested GIII and GI viruses might have different efficiency to generate infectious particle [22] or the stability difference of NS3 protein [23] in infected CTR209 cells. In addition, the viability of GIII and GI virus-infected CTR209 cells was non-significantly different at 28°C and 34°C (S2 Fig).

**Fig 2:**
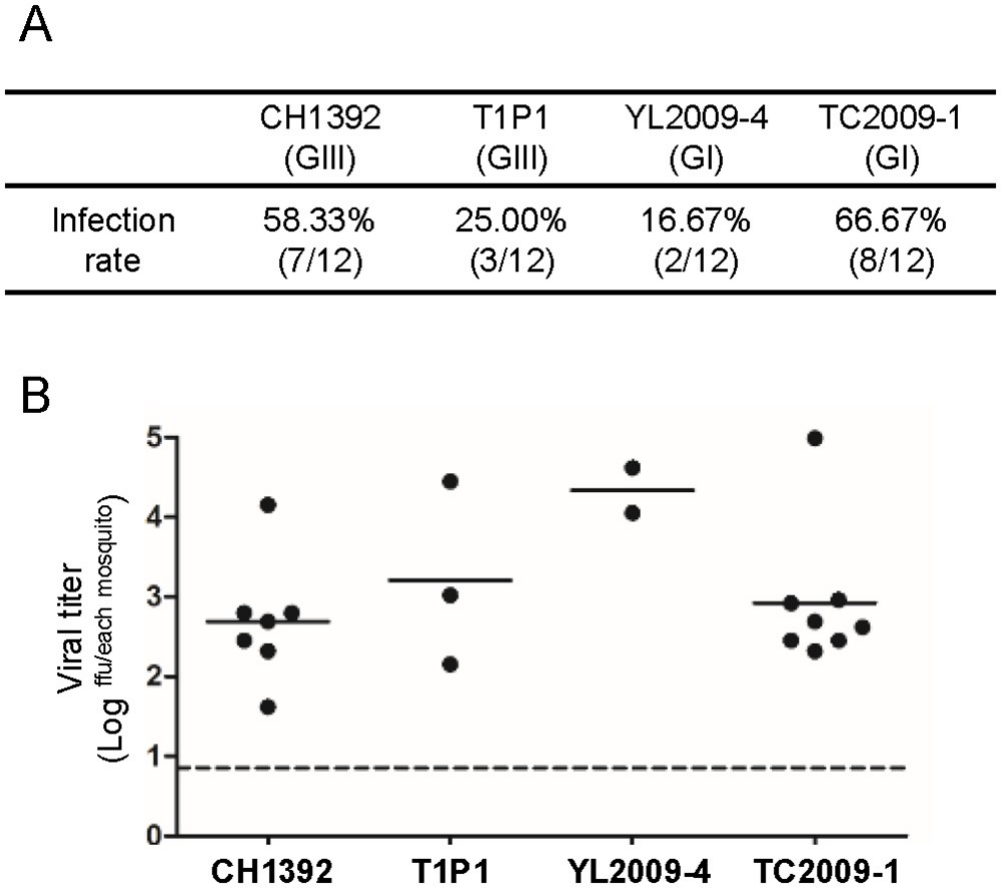
The indistinguishable infectivity of GIII and GI JEVs in *Culex tritaeniorhynchus*. Female *Culex tritaeniorhynchus* mosquitoes (n = 12 per group) were fed with the pig blood meal mixed with 8×10^6^ ffu/ml of JEVs (CH1392, T1P1, YL2009-4, or TC2009-1). (A) The viral infection rate was calculated by the number of JEV-positive mosquitoes divided by the number of feeding mosquitoes. (B) The viral titer (ffu) for each whole mosquito body was detected by micro-antigen focus assay at 14 days post-feeding. Each dot represents an individual mosquito. The mean value is shown as a horizontal line. Statistical analysis was performed by one-way ANOVA followed by Turkey’s Multiple Comparison Test.

To analyze viral replication in mosquitoes *in vivo*, we fed female *Culex tritaeniorhynchus* mosquitoes with pig blood mixed with 8x10^6^ focus forming units (ffu) of GIII or GI JEVs. We determined the infection rate by detection of viral titer in each mosquito 14 days post-infection (DPI). GIII CH1392 virus and GI TC2009-1 virus had higher infectious rates (58.33% and 66.67%) than GIII T1P1 virus and GI YL2009-4 virus (25.00% and 16.67%) whereas no genotypic differences were observed in infectivity or in viral titer among positive mosquitoes (Fig 2). These *in vitro* and *in vivo* results suggest that the *Culex tritaeniorhynchus* mosquito vector may play a minor role in genotype replacement.

### Host and/or the temperature-dependent replication advantage of GI virus over GIII virus

**Fig 3:**
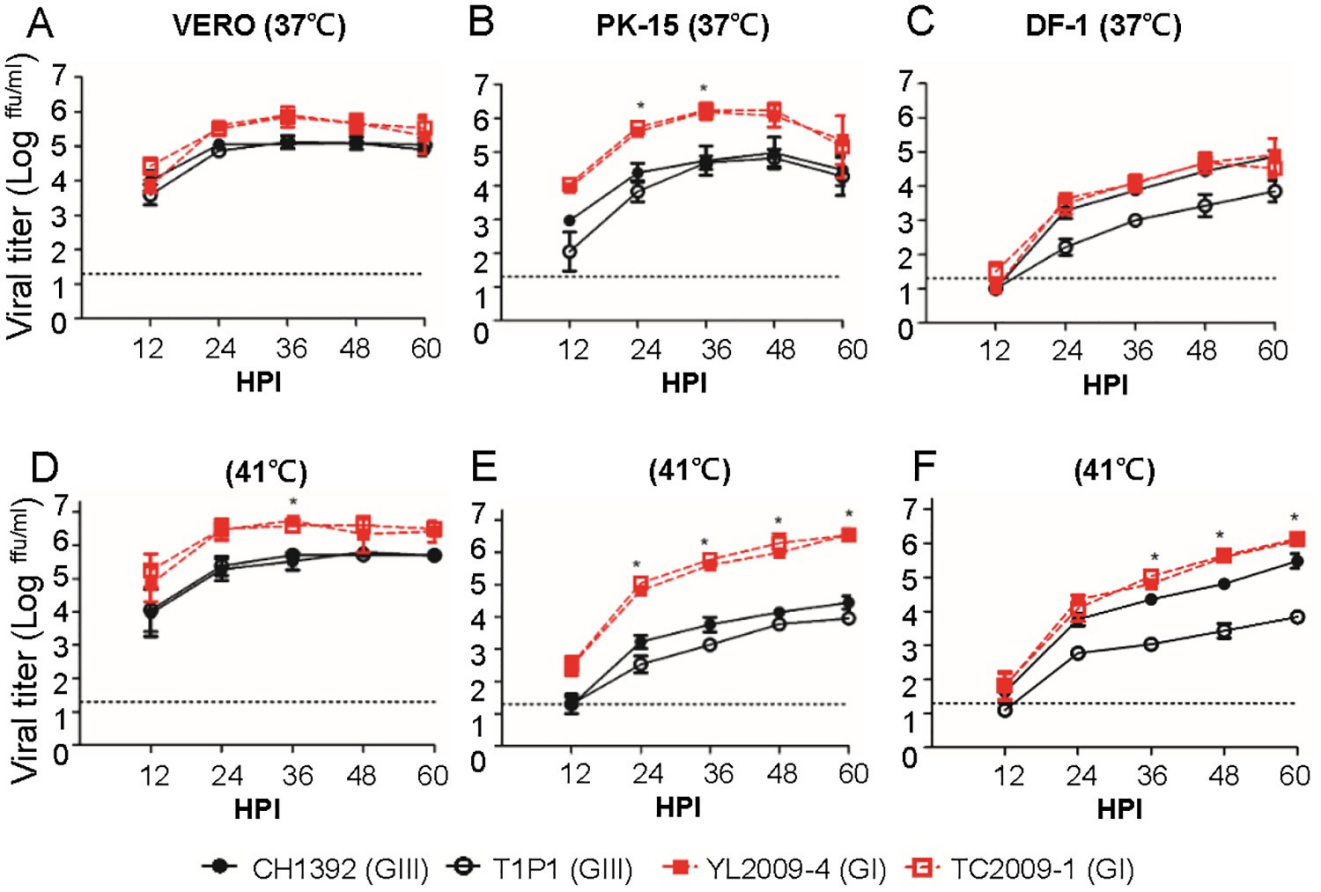
The replication advantage of GI JEVs is due to the amplifying host and/or the replication temperature. (A-F) GIII CH1392 virus (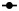), GIII T1P1 virus (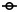), GI YL2009-4 virus (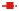), and GI TC2009-1 virus (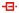) were inoculated onto VERO (A and D), PK-15 (B and E), and DF-1 cells (C and F) at 0.5 MOI and incubated at 37°C (A-C) or 41°C (D-F). The viral titer (ffu/ml) was determined in the supernatant at 12, 24, 36, 48, 60 HPI by micro-antigen focus assay. Mean with SEM for the triplicates is shown. The difference in viral titer was analyzed with one-way ANOVA followed by Turkey’s Multiple Comparison Test. A significantly genotype-specific difference is indicated by an asterisk (P< 0.05).

To evaluate the role of amplifying hosts in genotype replacement, we compared the *in vitro* replicative ability of GIII CH1392 and T1P1 strains to GI YL2009-4 and TC2009-1 strains in pig- and poultry-derived cells (PK-15, DF-1, CER, and DE cells) and primate-derived control cells (VERO cells) at 37°C (Fig 3A–3C, S3A and S3B Fig). A similar growth curve of GIII and GI viruses was observed in VERO cells (Fig 3A); in contrast, GI YL2009-4 and TC2009-1 viruses produced 1–2 -log higher viral titers than GIII CH1392 and T1P1 viruses in PK-15 cells at 24 and 36 HPI (p< 0.05) (Fig 3B). In poultry-derived cells (DF-1, CER, DE cells), two GI viruses and GIII CH1392 virus replicated to similar viral titers but their viral titers were higher than GIII T1P1 virus (Fig 3C, S3A and S3B Fig). Natural amplifying hosts for JEV have a higher body temperature of 40–44°C in avian species and 38–40°C in pigs. In pigs, fever can be as high as 41°C following JEV infection [24]. Therefore, we inoculated JEVs into VERO, PK-15, and poultry-derived cells (DF-1, CER, DE cells) and analyzed viral replication at 41°C. Interestingly, as the temperature increased, the overall viral titers of GI viruses were significantly higher than GIII viruses in amplifying host-derived cells and at the single time point of 36 HPI in VERO cells (p< 0.05) (Fig 3D–3F, S3C and S3D Fig). An apparently higher relative intensity of NS3 proteins was also observed at 48 HPI in GI virus-infected PK-15 and DF-1 cells but not in VERO cells at 41°C (S1G–S1L Fig). However, the ratio of NS3 to β-actin was inconsistent with extracellular infectious particles in the GIII virus-infected DF-1 cells. This suggested two GIII viruses might have different efficiency to generate infectious particle [22] or the stability difference of NS3 protein [23] in infected DF-1 cells.

The higher thermal stability at elevated temperature for GI viruses could be a contributing factor related to enhancement of viral replication of GI viruses. To determine the influence of viral thermal stability, we incubated equal amounts of GIII and GI viruses at 37°C and 41°C, and examined residual viral titers at 3, 6, 9, and 12 hours post-treatment (HPT) (S4 Fig). The viral infectious rates of both genotypes dropped to less than 50% after 3-HPT at 37°C and even more at 41°C (S4A and S4B Fig). There was no statistical difference between the half-life of infectivity of GIII and GI viruses at both 37°C and 41°C (S4C Fig). Besides, there was no genotypic difference on cell viability (VERO, PK-15, and DF-1) after GIII and GI virus infection except lower viability of GI virus-infected PK-15 cells, producing higher viral tier, at 41°C at 60 HPI (p< 0.05) (S2 Fig). These results suggest that the replication advantage of GI virus over GIII virus occurred in an amplifying host but was independent of external viral thermal stability and virus-infected cell viability.

### Experimental GI virus infection induced a higher and earlier viremia in pigs and poultry

**Fig 4:**
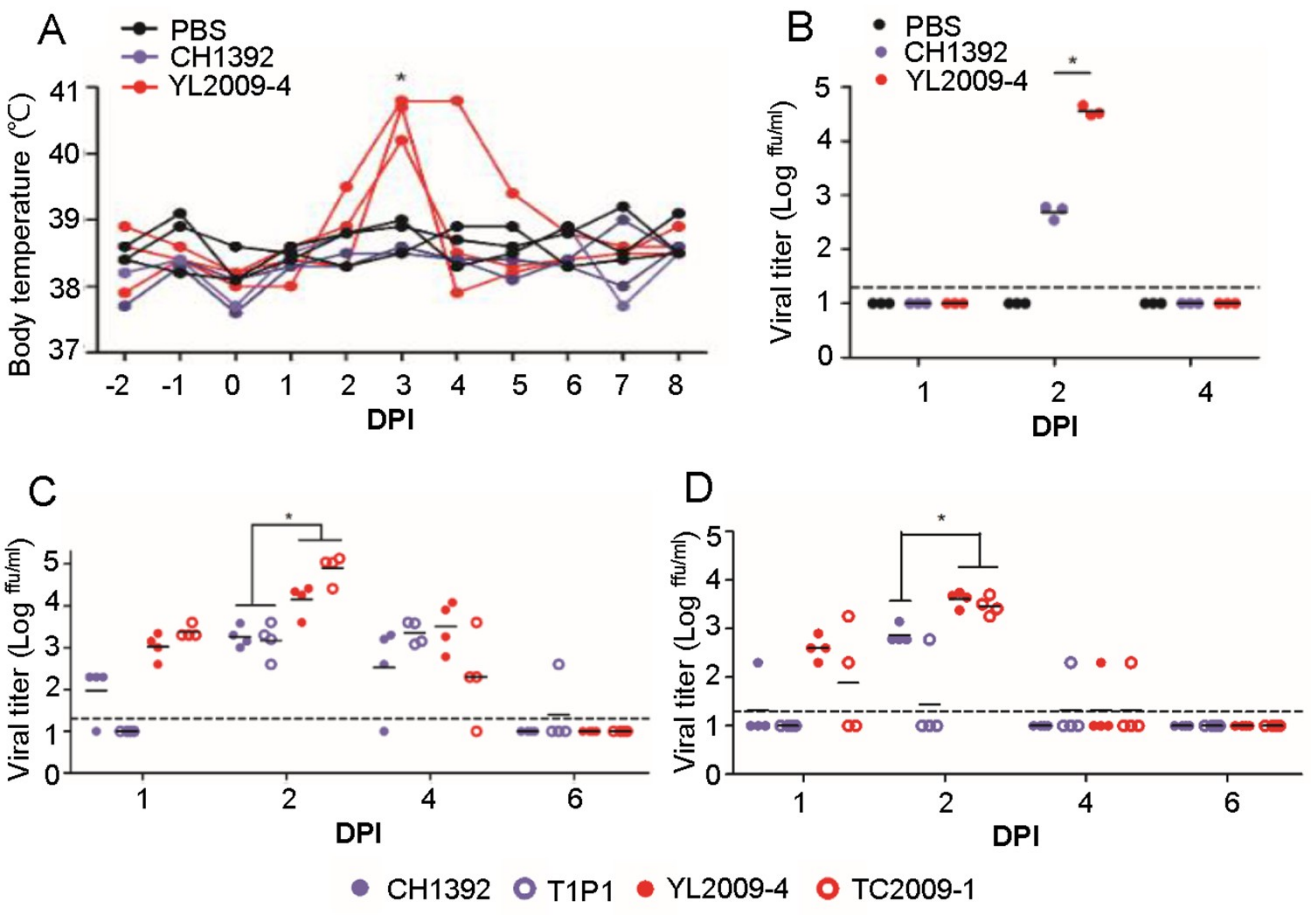
Comparison of viremia in experimentally-infected pigs and poultry using GI and GIII JEVs. (A and B) Nine specific pathogen free (SPF) pigs (n = 3 per group) were subcutaneously inoculated with 10^5^ ffu of GIII CH1392 virus (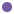) or GI YL2009-4 virus (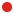), or PBS (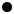). (A) The change of each pig’s body temperature before and after viral infection is shown. (B) The viral titer in pig plasma was detected by micro-antigen focus assay at 1, 2, and 4 days post infection (DPI). (C and D) One-day old chickens (n = 4 per group) and two-day old ducklings (n = 4 per group) were subcutaneously injected with 10^4^ ffu of GIII CH1392 (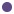), GIII T1P1 (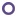), GI YL2009-4 (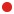), and GI TC2009-1 viruses (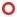). The viral titer in plasma recovered from JEV-infected chickens (C) or ducklings (D) at 1, 2, 4, and 6 DPI (n = 4 per group) was determined by micro-antigen focus assay. The detection limit is indicated as a dotted line. (A-D) A dot plus a horizontal line represent an individual animal and a mean viral titer (C, D) of the group, respectively. The statistics comparing either two or four viruses were determined by a Student’s Two-Tailed *t*-test or one-way ANOVA followed by Turkey’s Multiple Comparison Test. The statistical difference was noted by an asterisk (P< 0.05).

Enhancement of GI virus replication ability in pig- and poultry-derived cells was further investigated *in vivo*. JEV–infected pigs exhibited either an asymptomatic manifestation or developed fever with sufficiently high viremia to ensure the transmission of virus by engorged mosquitoes [8]. We subcutaneously inoculated 10^5^ ffu of GIII CH1392 virus, GI YL2009-4 virus, and or PBS into ten-week old, specific-pathogen free (SPF) pigs (three SPF pigs per group), and then monitored daily body temperature and viremia. At 3 DPI, three GI virus-infected pigs developed fever averaging 40.6°C while GIII virus-infected and PBS-inoculated pigs maintained normal body temperature until euthanasia at 8 DPI (Fig 4A). No detectable viremia was observed in the infected pigs at 1-DPI. A significantly higher viremia was detected the following day in GI YL2009-4 virus-infected pigs with an average viral titer of 10^4.6^ ffu/ ml compared to GIII CH1392 virus-infected pigs with a viral titer of 10^2.7^ ffu/ml (p< 0.05). However, viremia was undetectable at 4-DPI in all virus- and PBS-inoculated pigs (Fig 4B). The higher viremia in GI virus-inoculated pigs was consistent with the higher RNAemia detected in the GI virus-infected pigs at 2 DPI (p< 0.05) (S5A Fig). Although only a limited number of SPF pigs were used in this study, we found that GI YL2009-4 virus infection induced a higher viremia and higher fever than GIII CH1392 virus at 3 DPI.

Viremic migratory birds are suspected of spreading GI viruses between countries but the role of avian species in local transmission of GI virus has never been determined [14]. Experimentally JEV-infected young chickens and ducklings showed an age-dependent ability to induce sufficient viremia for mosquito infection [25]. To compare the replication ability of GIII to GI virus in domestic avian species, we subcutaneously inoculated 10^4^ ffu of GIII CH1392 and T1P1 strains, GI YL2009-4 and TC2009-1 strains or PBS into one-day old chickens (Fig 4C) and two-day old ducklings (Fig 4D). As early as 1 DPI, we found that 100% (8/8) and 75% (6/8) of GI virus-infected chickens and ducklings developed viremia, while 37.5% (3/8) and 12.5% (1/8) of GIII virus-infected chickens and ducklings, respectively, developed viremia (Fig 4C and 4D). GIII and GI virus titers were highest at 2 DPI and subsequently dropped the following 2 days (Fig 4C and 4D). These results showed that GI viruses replicated to a significantly (p<0.05) higher viremia (0.60–1.73-log higher) as well as earlier and longer lasting than GIII viruses in chickens and ducklings. The higher viremia was supported by the higher RNAemia detected in GI virus-infected poultry compared to GIII virus-infected poultry at 2 DPI (p< 0.05) (S5B and S5C Fig). However, no clinical signs were observed in the JEV-infected and PBS-inoculated poultry during the 6-day observation period.

### Nonstructural proteins 1 to 3 (NS1-3) determine the replication advantage of GI virus in host-derived cells

**Fig 5:**
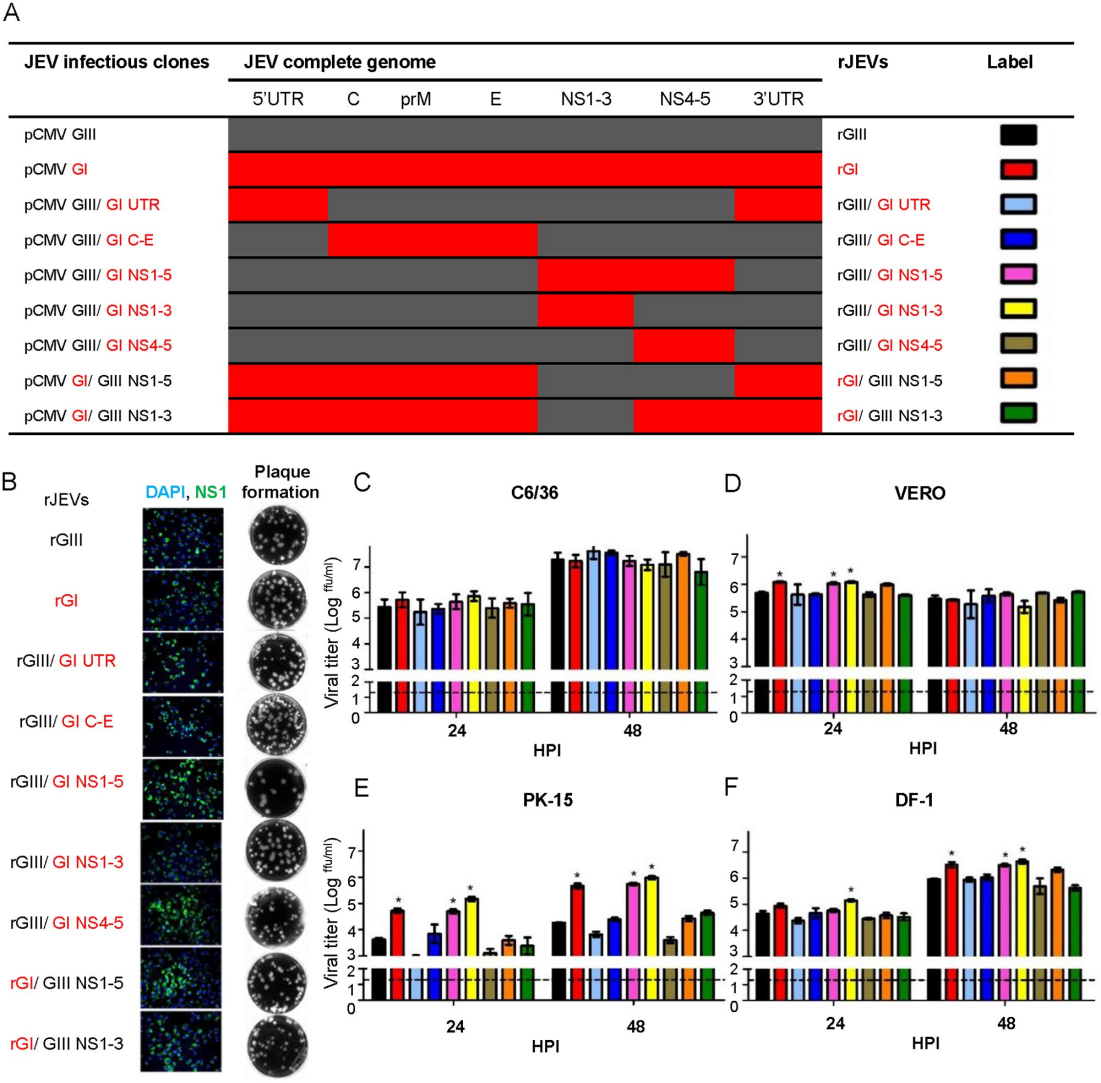
NS1-3 genes determine the major replication advantage of rGI virus in amplifying host-derived cell lines. (A) The schematic diagram displays viral genes encoded in the infectious clones and rJEVs. GIII virus- and GI virus-derived genes are shown in dark gray and red, respectively. (B) The infectious clones were transfected into BHK-21 cells and incubated for 3 to 4 days. The replication of rJEVs was detected in the cells by immunofluorescence using mouse anti-JEV NS1 antibodies and Alexa Fluor 488-labeled goat anti-mouse IgG antibodies. The cellular nucleus was localized by DAPI staining. The secretion of rJEVs was analyzed by the plaque assay in BHK-21 cells. (C-F) The rJEVs were inoculated onto C6/36 (C), VERO (D), PK-15 (E), and DF-1 (F) cells at 0.5 MOI, and incubated at 28°C (C6/36) or at 41°C (VERO, PK-15, DF-1). Supernatants were collected from each rJEV-infected cell culture at 24 and 48 HPI, and viral titers determined by micro-antigen focus assay. Mean with SEM for triplicates is shown. A horizontal dotted line indicates the detection limit. The statistical analysis was conducted with one-way ANOVA followed by a post test using Dunnett’s Multiple Comparison Test using rGIII virus as a control. An asterisk indicates that rJEV has a significantly higher titer than rGIII virus (P< 0.05).

To investigate the viral genetic determinants for the enhancement of GI virus infectivity, we initially analyzed the amino acid variances between seventy-one GIII viruses and seventy-seven GI viruses. Nine and twenty-four GI virus-specific and highly consensus substitutions were identified in structural and non-structural proteins, respectively, and three were non-conservative, charge-altering substitutions (Table 1). These substitutions as well as the other forty-three variations were observed between GIII and GI viruses used in this study. We also identified the cyclization structural variants formed by GIII and GI viruses in the untranslated region (UTR, S6 Fig), which might influence viral RNA synthesis [26]. To pin-point the target gene, we constructed five infectious clones of derived chimeric viruses (pCMV GIII/GI UTR, pCMV GIII/GI C-E, pCMV GIII/GI NS1-5, pCMV GIII/GI NS1-3, pCMV GIII/GI NS4-5) and one GI virus infectious clone (pCMV GI) by introducing the variant region of GI virus into the GIII virus infectious clone (pCMV GIII) (Fig 5A). These infectious cDNA clones were transfected into BHK-21 cells and subsequently detected by the expression of viral NS1 protein using an immunofluorescence assay (Fig 5B). Recombinant viruses, recovered from the transfected cells, formed similar sized plaques in BHK-21 cells (Fig 5B). Next we analyzed the replication ability of five chimeric recombinant viruses (rGIII/GI UTR, rGIII/GI C-E, rGIII/GI NS1-5, rGIII/GI NS1-3, and rGIII/GI NS4-5), recombinant GIII (rGIII) virus, and recombinant GI (rGI) virus in C6/36 cells at 28°C and in vertebrate cells (VERO, PK-15, and DF-1) at 41°C (Fig 5C–5F). All recombinant viruses reached similar titers in the range of 10^7.09^ to 10^7.62^ ffu/ml in C6/36 cells at 48 HPI. However, rGI virus exhibited a robust replication and significantly higher titer than rGIII virus in VERO (10^0.6^-fold difference at 24 HPI, p< 0.05), PK-15, (10^1.1^ and 10^1.4^-fold differences at 24 and 48 HPI, p< 0.05) and DF-1 cells (10^0.6^-fold difference at 48 HPI, p< 0.05) (Fig 5D–5F). Interestingly the rGIII/GI NS1-5 and rGIII/GI NS1-3 viruses replicated as efficiently as rGI virus, and produced a significantly higher titer than rGIII virus in VERO, PK-15, and DF-1 cells (p< 0.05). In contrast, rGIII/GI UTR, rGIII/GI C-E, rGIII/GI NS4-5, and rGIII viruses all maintained a low replication efficiency. These results demonstrated that the major genetic determinants for the enhancement of GI virus infectivity are located within NS1-3 proteins.

**Table 1:** The amino acid differences between GI and GIII JEVs.

Viral proteins	Consensus substitutions of GI JEV viral proteins^a^	Strain-specific substitutions
Capsid	**K70R**, **R100K**, **G110S**, **V120I**, *I122T*	K10R^f^, Q102R^d^,
prM	**T57A**, **M/A58V**,	M44L^d^, S90T^d^, V140T^e^/A^f^, N148T^b^^,^^c^, N149T^b^^,^^c^/S^e^^,^^f^
E	*T129M*, **A222S**, **S327T**, **A366S**,	S123N^f^, K209R^b^^,^^c^, V385I^d^
NS1	**K51Q**, **A70S**, **H147R**, **Y206L**, **K251R**, **V298I**	S175N^f^,
NS2A	**V6I**, **T97A**, **S149T**, *T151A*, **K187R**	S70N^f^, V154I^f^,
NS2B	*E55D*, **D65E**, **V99L**	K78N^e^, D81E^b^^,^^c^^,^^d^
NS3	*S14L*, **A78S**, **A105P**, **E177D**, **N182S**, *R185K*, *E354D*	K15R^f^, I109F^b^, E122K^c^, V123I^e^, Q188R^f^, R218K^d^, R237K^e^
NS4A	**I110V**	
NS4B	**K20R**, **N73S**, *A118V*	S24P^f^, F88S^d^, A205R^d^, A216R^d^
NS5	**E22D**, **R101K**, **K280R**, *K287R*, *A372V*, *D429G*, *D438N*, **R432L**, **E588G**, *V878I*	A338V^e^, Y^b^^,^^c^^,^^d^/H386H^e^^,^^f^, W751C^b^^,^^c^, D776Y^d^

^a^A total of 71 GIII and 77 GI viruses were analyzed by VisSPA software. These conserved substitutions were observed for 70% or 95% of GIII and GI JEVs used in this study, and were respectively highlighted in italic or boldface. Substitutions resulting in an altered charge were underlined.

^b^GIII CH1392 strain-specific substitutions

^c^GIII T1P1 strain-specific substitutions

^d^GIII RP9 strain-specific substitutions

^e^GI YL209-4 strain-specific substitutions

^f^GI TC2009-1 strain-specific substitutions

Conversely, we investigated the influence of the replication ability of GIII virus- derived NS1-5 and NS1-3 proteins on the rGI backbone for amplification in host-derived cells. The chimeric clones pCMV GI/GIII NS1-5 and pCMV GI/GIII NS1-3 were constructed to produce rGI/GIII NS1-5 and rGI/GIII NS1-3 viruses as described above (Fig 5A and 5B). The recombinant viruses yielded similar titers compared to rGIII virus but produced significantly lower titers than rGI virus in VERO, PK-15, and DF-1 cells at 41°C (p< 0.05) (Fig 5D–5F). These results further support the conclusion that GI-derived NS1-3 genes made a major contribution to enhanced replication of GI virus in host-derived cells at elevated temperature.

### rGIII/GI NS1-3 virus induced higher viremia than rGIII virus in pigs and chickens

**Fig 6:**
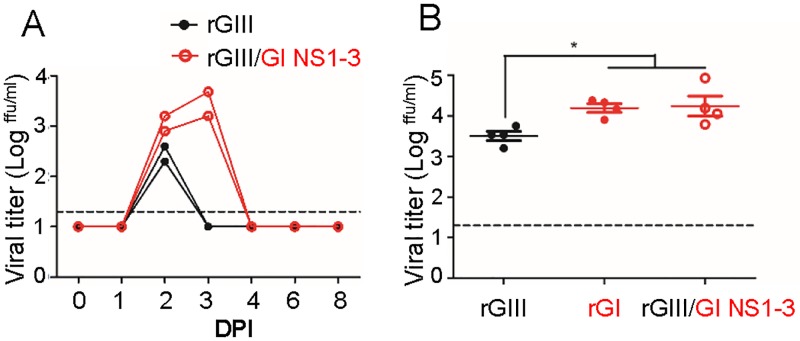
Enhancement of GI virus NS1-3 proteins in infected pigs and chickens. 10^5^ and 10^4^ ffu of rGIII (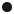), rGI (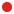), and rGIII/ GI NS1-3 viruses (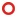) were subcutaneously inoculated into SPF pigs (A) and one-day old chickens (B). The viral titer (A and B) in plasma collected from the infected pigs at days 0 to 8, and from the infected chickens at 60 HPI, were detected by micro-antigen focus assay. Each dot represents an individual animal. A horizontal dotted line indicates the detection limit. Mean with SEM is shown. The viremia in chicken plasma was statistically analyzed with one-way ANOVA followed by Turkey’s Multiple Comparison Test. A significant difference is indicated by an asterisk ([B], P< 0.05).

To verify the role of NS1-3 genes *in vivo*, we compared the replication ability of rGIII/GI NS1-3 virus to rGIII virus by subcutaneously inoculating 10^7^ ffu and 10^4^ ffu of the viruses into ten-week old SPF pigs and 1-day old chickens, respectively (Fig 6). The rGIII and rGIII/GI NS1-3 virus-infected pigs developed viremia with an average titer of 10^2.45^ and 10^3.05^ ffu/m at 2 DPI. The following day, rGIII/GI NS1-3 virus-infected pigs reached peak viremia but no viremia was detected in rGIII virus-inoculated pigs (Fig 6A). This difference was also observed in the plasma viral RNA collected from the infected pigs at 2 DPI (S7A Fig). These results highlighted the higher and extended viremia in rGIII/GI NS1-3 virus-infected pigs compared to rGIII virus-infected pigs. In addition, we also observed that rGIII/GI NS1-3 virus induced significantly higher viremia and RNAemia in chickens with 5-fold and 6-fold increases in viral titer and viral RNA compared to rGIII virus (p< 0.05) at 60 HPI, respectively (Fig 6B and S7B Fig). These results further suggest that the major viral determinants for the enhancement of GI virus infectivity in pigs and chickens were located on NS1-3 proteins.

### The replication advantage of rGIII/GI NS1-3 virus was associated with NS2B/NS3 mutations

**Fig 7:**
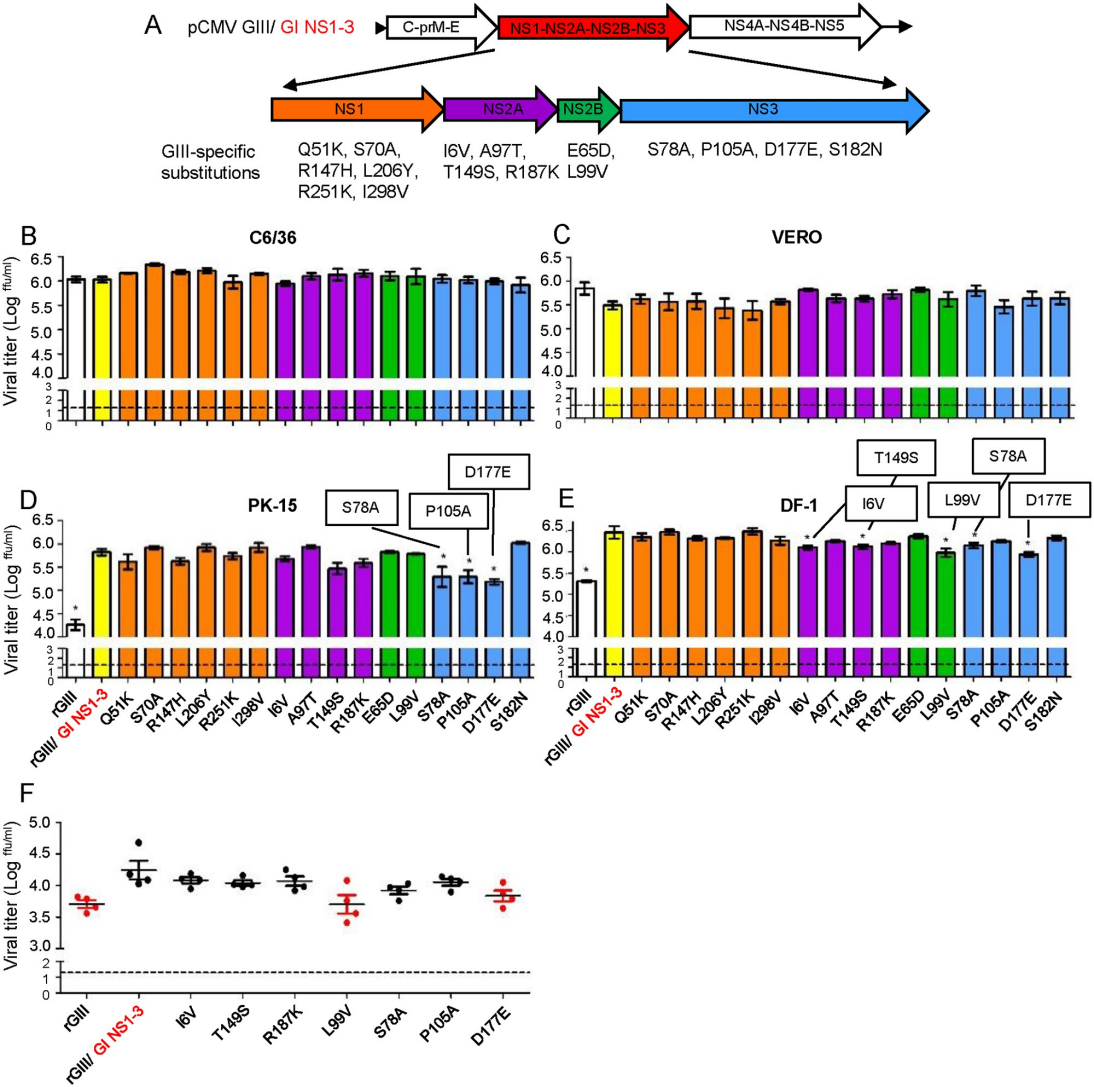
Effect of introduction of GIII virus-specific NS2B/NS3 substitutions into GI virus. (A) The schematic diagram shows the location of sixteen GIII virus-specific substitutions on NS1-3 proteins. (B-E) The sixteen mutant rGIII/ GI NS1-3 viruses encoding single NS1, NS2A, NS2B, or NS3 substitutions are respectively colored with orange, purple, green, and blue. The rGIII and rGIII/ GI NS1-3 virus controls are shown colored with white and yellow. These rJEVs were infected into C6/36 (B), VERO (C), PK-15 (D), and DF-1 (E) cells at 0.5 MOI and replicated at 28°C (C6/36) or 41°C (VERO, PK-15, and DF-1). The viral titer in each supernatant was detected at 48 HPI by micro-antigen focus assay. Mean with SEM for the triplicate samples is shown. (F) 10^4^ ffu of the selected rJEVs were subcutaneously inoculated into one-day old chickens (n = 6 per group). The viral titer in collected plasma was detected at 60 HPI and measured by micro-antigen focus assay. A horizontal dot line represents an individual animal and mean of a group respectively. Error bars indicate SEM. A horizontal dot line indicates the detection limit. The statistical analysis was determined by one-way ANOVA followed by Dunnett’s Multiple Comparison Test utilizing rGIII/ GI NS1-3 virus as a control. A significant difference is indicated by an asterisk (B-E) or shown as a red dot (F) (P< 0.05).

To verify the specific substitution(s) of GI NS1-3 proteins involved in the enhancement of GI replication, we conducted a loss-of-function experiment by introduction of a single GIII virus-specific and highly consensus substitution for NS1-3 proteins of the rGIII/GI NS1-3 chimeric virus instead of the rGI virus (Fig 7A). The influence of these substitutions on the replication ability of rGIII/GI NS1-3 virus was evaluated in C6/36 cells at 28°C or in VERO, PK-15, and DF-1 cells at 41°C. As expected, the replication advantage of the rGIII/GI NS1-3 virus was consistently observed in PK-15 and DF-1 cells but not in C6/36 and VERO cells as compared to the rGIII virus at 48 HPI (Figs 5 and 7). However, the enhanced replication of the rGIII/GI NS1-3 virus was significantly reduced after the introduction of three single-substitutions (NS3-S78A, NS3-P105A, or NS3-D177E) in PK-15 cells (p< 0.05) and five single-substitutions (NS2A-I6V, NS2A-T149S, NS2B-L99V, NS3-S78A, or NS3-D177E) in DF-1 cells (p<0.05), respectively. The NS2A-R187K substitution had a minor effect on viral titer in both cell lines (p> 0.05) (Fig 7D and 7E). These results suggest that NS1 substitutions might not be critical factors for influencing the phenotype of the rGIII/GI NS1-3 virus.

The loss-of-function experiments were also used to evaluate substitutions for the degree of influence on the *in vivo* replication ability of rGIII/GI NS1-3 virus in 1-day old chickens. The rGIII and parental rGIII/GI NS1-3 viruses were inoculated as infection controls (Fig 7F and S8A Fig). As expected, rGIII/GI NS1-3 virus replicated to a significantly higher viremia than rGIII virus, and yielded 10^4.25^ ffu/ml (Fig 7F) and 10^6.93^ viral RNA copies/ml (S8A Fig) in the chickens at 60 HPI. In contrast, the recombinant viruses encoding a single NS2B-L99V, NS3-S78A, or NS3-D177E substitution induced a significantly lower viremia or RNAemia than the parental rGIII/GI NS1-3 virus (p< 0.05). The other recombinant viruses encoding a single NS2A-I6V, NS2A-T149S, NS2A-R187K or NS3-P105A substitution showed a viremia comparable to rGIII/GI NS1-3 virus in chickens. These results further supported a conclusion that the residues NS2B-99, NS3-78, and NS3-177 were involved in the replication enhancement of GI virus *in vitro* and *in vivo*.

### The contribution of GI NS2B-V99L and NS3-A78S/E177D to the enhancement of GI virus infectivity

**Fig 8:**
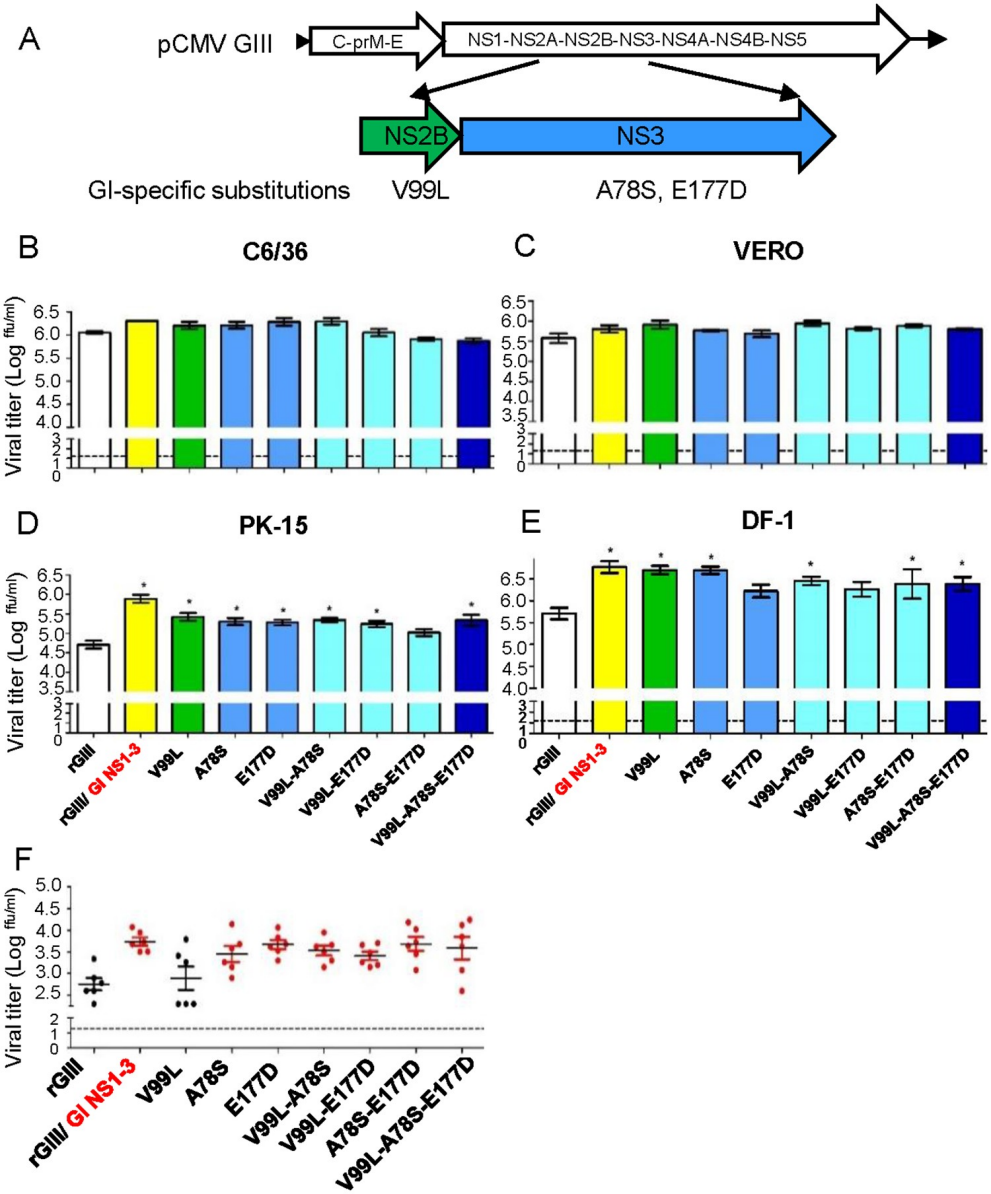
The contribution of NS2B/NS3 substitutions on replication advantage of GI virus in amplifying hosts. (A) The schematic diagram displayed the location of GI virus-specific NS2B/NS3 substitutions. (B-E) The single, double, or triple substitutions were introduced into NS2B/NS3 proteins of rGIII viruses. Single NS2B or NS3 substitutions with green or blue color. Double or triple substitutions with cyan or dark blue color. The rGIII, rGI, and rGIII/ GI NS1-3 viruses with white, red, and yellow color. The mutant rGIII viruses infected C6/36 (B), VERO (C), PK-15 (D), and DF-1 (E) cells at 0.5 of MOI and replicated at 28°C (C6/36) or 41°C (VERO, PK-15 and DF-1). The viral titer in the supernatant was detected at 48 HPI by the micro-antigen focus assay. Mean with SEM of the triplicates was displayed. (F) 10^4^ ffu of the rJEVs were subcutaneously inoculated into one-day old chickens (n = 6 per group). The viral titer in plasma was detected at 48 HPI by the micro-antigen focus assay. A dot and horizontal line represent an individual animal and mean of a group. Error bars indicate SEM. A dotted line indicates the detection limit. The statistical analysis was calculated with one-way ANOVA followed by Dunnett’s Multiple Comparison Test utilizing the control of rGIII virus. A significant difference was indicated as an asterisk (B-E) or shown as a red dot (F) (P< 0.05).

To investigate the inter-dependency among the substitutions, we introduced single or multiple GI virus NS2B-V99L, NS3-A78S, and NS3-E177D substitutions into rGIII viruses (Fig 8A). Seven mutant rGIII viruses were generated and their infectivity evaluated using C6/36 cells at 28°C and VERO, PK-15, and DF-1 cells at 41°C. rGIII, rGI, and rGIII/GI NS1-3 viruses were included as controls (Fig 8B–8E). As expected, all recombinants yielded similar viral titers in C6/36 and VERO cells but rGIII virus exhibited a significantly lower titer than rGIII/GI NS1-3 viruses in PK-15 and DF-1 cells at 48 HPI. Mutant rGIII viruses encoding single NS2B-V99L, NS3-A78S, double NS2B-V99L-NS3-A78S, or triple substitutions yielded significantly higher viral titers (0.59 to 1.00-log increase in viral titer) in both PK-15 and DF-1 cells as compared to rGIII virus (p< 0.05). The effect of substitutions on viral replication was further evaluated by inoculating mutant rGIII viruses into 1-day old chickens. rGIII/GI NS1-3 virus replicated to higher titer (1.02 log) (Fig 8F) and produced higher levels of viral RNA (0.99 log) (S8B Fig) than rGIII virus at 48 HPI. With the exception of the NS2B-V99L substitution, the remaining mutant rGIII viruses encoding single and multiple substitutions had 0.68 to 0.96-log higher viral titers and 0.46 to 1.01-log higher viral RNA production than rGIII virus in 1-day old chicken. However, we were unable to detect an apparent synergistic effect among three substitutions.

## Discussion

Emerging GI virus has gradually replaced GIII virus as the dominant JEV isolated from human cases, stillborn piglets, and *Culex tritaeniorhynchus* since the 1990s. The mechanism of genotype replacement remains unclear, especially the role of the genetic determinants affecting the local pig-*Culex tritaeniorhynchus* transmission cycle. In this study, we identified the contribution of NS2B/NS3 mutations correlated with enhanced replication of GI virus in amplifying hosts: domestic pigs as well as in day-old chickens and two-day old ducklings. The role of *Culex tritaeniorhynchus* mosquito might be less significant in the genotype replacement.

There are two geographic variants of GI JEVs: viruses from GI-a clade are mainly distributed in tropical areas of Asia and Australia [13, 16, 27] and viruses of GI-b clade used in this study are widely distributed in southern and eastern Asia [16]. Previous studies have suggested that GI-a or GI-b viruses can compete with GIII for the same mosquito vector and amplifying hosts but are less likely to co-circulate in the same geographic locations [13, 20, 27, 28]. Mosquito vectors play a critical role in the occurrence of newly emerging flavi- and alphaviruses [29–32]. The previous reports indicated that GI-b virus replicated to higher titer in *Aedes albopictus* mosquito-derived cells [18] but inconsistent results on the infectivity of GI-a, GI-b, and GIII viruses were observed in *Culex quinquefasciatus* [33, 34]. *Culex tritaeniorhynchus* is the primary mosquito vector for GI and GIII viruses and account for 93.58% and 74.22% of mosquito-derived isolates, respectively [20]. We used *in vitro Culex tritaeniorhynchus*-derived cells as well as *in vivo Culex tritaeniorhynchus* mosquitoes to study the infectivity of GI-b and GIII viruses in the current study. Our study results indicated that both genotypes of JEV replicated to similar viral titers regardless of the assay system used (Fig 2) and suggested that the primary vector, *Culex tritaeniorhynchus* mosquito, for GI-b and GIII viruses is less likely to play a significant role in the genotype replacement of GIII to GI-b.

Migratory birds were suspected of spreading GI-b virus from southern to southeastern Asia and thus associated with JEV genotype replacement [19, 35]. The ardeid birds (herons and egrets) and pigs play an important role in local transmission of JEV [8]. All host-derived GI viruses are isolated from pigs [20]. However, JEV can infect young domestic poultry (chickens and ducklings) which are suspected of being involved in local transmission of JEV due to viral titers being sufficiently higher than the minimum infectious dose for mosquito hosts [25]. Earlier or higher viremia in GI-b virus-infected birds [19] and ducklings [36] suggested that a domestic avian-mosquito cycle might enhance the transmission of GI viruses. GI-b virus induced higher viremia than GIII virus in pigs, young chickens and ducklings (Fig 4). However, previous studies showed similar replication ability of GI-b, JE-91 strain, isolated in 1990 and GIII virus in DF-1 cells and ducklings [18, 33]. We speculate that this difference could be a result of the genetic variation of earlier GI-b isolates and 3 days older ducklings used in the previous studies [18, 33]. The NS2B/NS3 protein sequences of GI and GIII viruses used in the previous studies were unavailable. Higher viremia induced by GI-b virus could enhance viral transmission by mosquitoes since the infection rate of JEV was dose-dependent in mosquitoes [37]. In addition, GI-b virus produced higher levels of viral RNA than GIII virus in pig tonsil and nasal mucosa explants, potentially enhancing oronasal transmission between pigs [38, 39]. Collectively, our study suggests that the replacement of GIII virus with GI-b virus as the dominant and circulating virus in the host-mosquito cycle is the result of enhancement of transmission efficiency in amplifying hosts, including pigs and domestic avian species, not in mosquito vectors.

**Fig 9:**
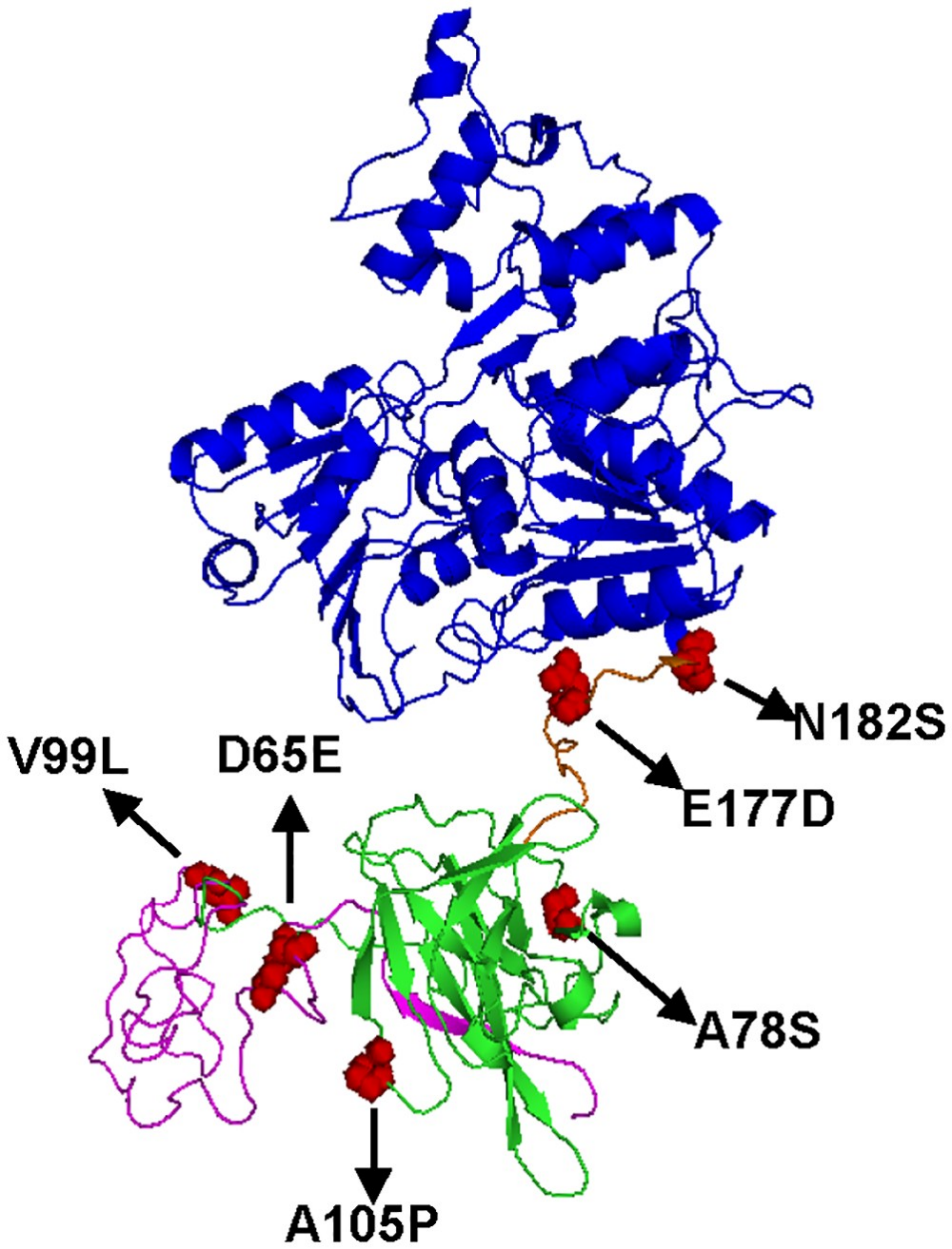
The conformational location of GI virus-specific substitutions on NS2B/NS3 proteins. (A) The homologous modeling of GI virus NS2B/NS3 proteins was carried out with SWISS-MODEL [47] by utilizing the template structure of MVEV NS2B/NS3 proteins (PDB: 2WV9). The 49 to 131 residues of NS2B protein, protease domain of NS3 protein, and the helicase domain of NS3 protein are highlighted with pink, green, and blue. The linker domain connecting proteinase and helicase domains of NS3 protein is colored orange. All GI virus-specific substitutions on NS2B/NS3 proteins are highlighted as red spheres.

Mutation in the NS1 protein of Zika virus and the NS3 protein of HCV has been shown to enhance viral fitness during flaviviral evolution [29, 40]. Genomic sequencing and analysis has suggested that substitutions in E, NS4B, and NS5 proteins were involved in the evolutionary advantage of GI virus [18, 20]. Experimental evidence provided in our study, however, suggested that the enhancement of GI-b virus infectivity in amplifying hosts was associated with NS2B/NS3 substitutions (Figs 7 and 8), especially the residues NS2B-99 and NS3-78 in the protease domain and NS3-177 in the loop connecting protease and helicase domains of NS2B/NS3 proteins (Fig 9). Other substitutions such as NS2B-D65E, NS2B-A105P, NS3-N182S, or NS1/NS2A only played a minor role in improving viral fitness. The GI virus NS2B-99, NS3-78, and NS3-177 residues were also observed in GII viruses or GV viruses (S2 Table). This implied the substitutions associated with genotype replacement of GIII virus by GI virus and geographical distribution of the other genotypes may be different. The NS2B/NS3 proteins were involved in viral RNA replication, polypeptide processing, and infectious particle assembly through enzymatic-dependent or -independent processes [41, 42]. Thus, the GI-b virus NS2B/NS3 substitutions may enhance viral replication at post viral entry, as supported by the observation that infectivity rates were similar between rGI and rGIII viruses in the infectious center assay (S9 Fig). Moreover, flaviviral NS2B/NS3 proteins harbor multiple strategies to evade host innate immunity [42, 43]. JEV NS2B/NS3 protease was able to cleave interferon stimulator [44]. This interferon antagonistic ability of JEV was critical for efficient replication and increased virulence in mice [45]. A novel interferon antagonist of NS1 protein has recently been identified in newly emerging Zika viruses associated with the current epidemics [46]. In contrast to PK-15 and DF-1 cells, the virus titer (Figs 7 and 8) and focus size (S10 Fig) had no significant influence by NS2B/NS3 substitution in interferon-deficient VERO cells, suggested that the virus titers and focus size may associate with interferon antagonism or other host factors. Therefore, we hypothesize those two possible mechanisms of enhancement in viral post-entry and innate immunity antagonistic ability result in the replication advantage of GI-b virus in amplifying hosts.

Flaviviruses adaptation to elevated temperatures have been shown to enhance fitness in avian species and mosquito vectors [21, 48]. The enhancement of GI-b virus infectivity in amplifying hosts was observed at elevated temperatures (Fig 3A–3F). A West Nile virus study found that thermostability of replication was associated with higher viremia in avian species [48]. The GI-b NS2B/NS3 substitutions might enable NS2B/NS3 protein complex or NS3/NS5 replicase complex to interact more effectively with heat shock proteins for proper folding and hence stability at elevated temperature [23, 49]. Thus, it is possible that higher body temperature or development of fever in amplifying hosts could positively modulate interferon activity of vertebrate hosts against viral infection [50, 51]. Therefore, the influence of temperature on enzymatic activity, heat shock proteins-interacting ability, and interferon antagonistic ability of GI-b and GIII virus NS2B/NS3 proteins should be investigated in future studies.

The study of GI-a and GIII virus infectivity in *Culex quinquefasciatus* were inconsistent in previous studies [33, 34]. The NS2B-99, NS3-78, and NS3-177 residues were conserved in GI-a and GI-b viruses but additional substitutions were identified on E-141, NS2A-105, and NS5-438. Thus, the virological factors underlying the replacement of GIII virus by GI-a virus may require re-evaluation of viral replication ability in both *Culex tritaeniorhynchus* and amplifying hosts using dominant, circulating GI-a isolates in the future.

There are two limitations of the current study: the limited number of SPF pigs used to reveal the replicative advantage of GI vs. GIII viruses and the lack of analytical data to determine the viral dissemination and transmission ability in *Culex tritaeniorhynchus*. However, even with these limitations we should not underestimate the disease burden of JEV caused by GI virus infection. We have no doubt that GI viruses are more efficiently transmitted in the amplifying host-mosquito cycle and have similar virulence compared to the GIII virus in human [52]. Thus, we suggest that it is important to continually monitor GI virus evolution and clarify the role of avian species in local transmission of GI virus.

## Materials and methods

### Ethics statement

Animal Use protocols were approved by the Institutional Animal Care and Use Committees (IACUCs) in National Chung Hsing University (NCHU) (protocol number: 102–107) and National Pingtung University of Science and Technology (NPUST) (protocol number: 104–013). All experimental protocols followed the Guide for the Care and Use of Laboratory Animals published by the National Institutes of Health.

A total of 196 1 day-old specific pathogen free (SPF) chickens (*Gallus gallus domesticus*) and 80 2 day-old minimum disease-free ducklings (*Cairina moschata*) were purchased from JD-SPF Biotech Co., Ltd and Livestock Research Institute (Council of Agriculture in Taiwan), respectively. These animals were kept in isolators at the avian holding facility in NCHU. Thirteen second-generation SPF pigs (Lee-Sung Strain) were purchased from the Agricultural Technology Research Institute in Taiwan, and housed in the negative air-pressure animal facility certified by the Association for Assessment and Accreditation of Laboratory Animal Care International (AAALAC) in the Animal Disease Diagnostic Center of NPUST, Taiwan. Avian and pig euthanasia performed by the use of CO_2_ inhalation and electrical stunning were approved by IACUCs in NCHU and NPUST, respectively.

### Cells and viruses

C6/36 cells (provided by Dr. Yi-Ling Lin from Academic Sinica, Taiwan) derived from the midgut of *Aedes albopictus* and CTR209 cells (provided by Dr. Kyoko Sawabe from National Institute of Infectious Disease, Japan) derived from embryos of *Culex tritaeniorhynchus* [53] were grown in Roswell Park Memorial Institute (RPMI) 1640 medium (Gibco) supplemented with 5% fetal bovine serum (FBS, Gibco) and in VP12 medium [54] supplemented with 10% FBS, respectively. Porcine kidney cells (PK-15, provided by Dr. Chienjin Huang from NCHU, Taiwan), chicken embryo fibroblast cells (DF-1, provided by Dr. Shan-Chia Ou from NCHU, Taiwan), chicken embryo related cells (CER) and duck embryo cells (DE and CER, provided by Dr. Poa-Chun Chang from NCHU, Taiwan), and monkey kidney cells (VERO, provided by Dr. Gwong-Jen J. Chang from Centers for Disease Control and Prevention (CDC), United States of America) were all grown in Dulbecco’s Modified Eagle Medium (DMEM, Gibco) supplemented with 10% FBS except for VERO cells with 5% FBS. Baby hamster kidney cells (BHK-21, provided by Dr. Wei-June Chen from Chang Gung University (CCU), Taiwan) were cultured in Minimum Essential Medium (MEM, Gibco) supplemented with 10% FBS. Mosquito cells or mammalian cells were maintained in incubators supplied with 5% CO_2_ at 28°C or 37°C. GIII subcluster II JE CH1392 and T1P1 viruses (provided by Dr. Wei-June Chen from CCU, Taiwan), and GI-b subcluster II JE YL2009-4 and subcluster I TC2009-1 viruses (from an already-existing collection in our lab) were used in this study. JEV strains CH1392, YL2009-4, and TC2009-1 were isolated from pools of field-captured *Culex tritaeniorhynchus* in 1990 and 2009, and T1P1 was isolated from the pool of *Armigeres subalbatus* in 1997 [55, 56]. All viral stocks were amplified in C6/36 cells and stored at -80°C until used.

### Growth kinetic curve

JEVs or rJEVs were inoculated onto C6/36, CTR209, PK-15, DF-1, CER, DE, and VERO cells at an MOI of 0.5 at 28°C for mosquito cells and at 37°C for mammalian cells. Infected cells were washed three times with 1X PBS after a one-hour incubation, and then subsequently incubated at 28°C or 34°C for mosquito cells and at 37°C or 41°C for mammalian cells. The supernatants from infected cells were collected and stored at −80°C until used. All experiments were conducted in triplicate, and the viral titers in the supernatants were determined by the micro-antigen focus assay.

### Micro-antigen focus assay

The micro-antigen focus assay was used to determine viral titers in the supernatants of infected cells, in the plasma recovered from infected animals, or in the supernatants of homogenized mosquitoes. Briefly, VERO cells were seeded in to 96-well plates at a cell count of 2.25×10^4^ cells/100 μL/well and incubated at 37°C, 5% CO_2_ overnight to allow a cell monolayer to form. The serially diluted samples were added into wells for one hour at 37°C. Infected VERO cells were overlayed with 1% methylcellulose mixed with DMEM medium supplemented with 2% FBS, and incubated at 37°C for 32 to 36 hours. The methylcellulose overlays were discarded by washing with 1X PBS, and the infected VERO monolayers were fixed with 75% acetone in PBS for 20 minutes. The fixed cells were air-dried and stained with mouse anti-JEV polyclonal antibody (provided by Dr. Gwong-Jen J. Chang from CDC, USA), followed by staining with horseradish peroxidase (HRP)-conjugated goat anti-mouse IgG antibody (Jackson ImmunoResearch, West Grove, PA). The foci were developed after the addition of Vector-VIP (Vector Laboratories, Burlingame, CA) into each well. The viral titer was calculated by the average number of foci-forming unit (ffu) per ml or per mosquito.

### Experimental infection of mosquitoes and animals

Laboratory-hatched female *Culex tritaeniorhynchus* mosquitoes were fed with 10% sucrose and maintained at 28°C. Mosquitoes were starved for 1 day prior to the blood-feeding experiment. The mosquitoes were fed *per os* with a JEV viremic blood meal, a mixture of pig blood cells and 8×10^6^ ffu/ml of JEV according to the previous study [57] and the stock titer of JEVs used in this study. The virus-infected mosquitoes were maintained in the different cage at 28°C. Infected mosquitoes were collected from cages by aspiration and homogenized individually at 14 days post- infection (DPI).

Thirteen ten-week old, JEV-seronegative SPF pigs were used to determine the replication ability of field-isolated JEVs (nine pigs) and rJEV (four pigs). SPF pigs were anesthetized with stresnil (China Chemical and Pharmaceutical Co., Ltd) and subcutaneously inoculated with PBS or 10^5^ ffu of GIII CH1392 virus, GI YL2009-4 virus, or 10^7^ ffu of rJEVs. Experimental pigs were monitored, the daily body temperatures were recorded, and clinical signs of infection were noted. Pig plasmas were recovered at different days post infection. All pigs were euthanized with electrical stunning at 8 DPI.

Eighty 1-day old chickens (16 per viral group) and eighty 2-day old ducklings (16 per viral group) were subcutaneously inoculated with one dose of PBS or 10^4^ ffu of GIII CH1392 virus, GIII T1P1 virus, GI YL2009-4 virus, or GI TC2009-1 virus. The daily activity and clinical signs of infection for chickens and ducks were monitored after JEV infection. We collected blood from four infected chickens or ducklings 1 day prior to infection and at 1, 2, 4, and 6 DPI. Plasma was mixed with anticoagulant at a final concentration of 0.33% sodium citrate (Sigma-Aldrich) in 0.85% sodium chloride (Sigma-Aldrich), and centrifuged at 3,000 rpm for 15 minutes. The ten-fold diluted plasma was recovered from the supernatant and stored at −80°C until used.

One hundred sixteen 1 day-old chickens (4 per group in Figs 6 and 7; 6 per group in Fig 8) were subcutaneously inoculated with PBS or 10^4^ ffu of rJEVs. The plasma was recovered from infected chickens at 48 or 60 HPI as described above, and stored at −80°C until used.

### Construction of the recombinant and site-directed mutated infectious clones

The infectious clone encoding the full genome of GIII JE RP9 virus was constructed using pBR322 plasmid and referred to as pCMV GIII (kindly provided by Dr. Yi-Ling Lin from Academic Sinica, Taiwan) in this study. JEV viral RNA was transcribed by CMV promoter and terminated by SV40 poly-A terminator. The precise JEV 3’ terminal sequence was generated by a ribozyme sequence of hepatitis delta virus (HDVr) incorporated right after the 3’UTR of JEV (5). To construct GIII and GI JEV chimeric infectious clones, we replaced five genetic fragments or the complete viral genome of pCMV GIII with the corresponding genes of GI JE YL2009-4 virus by blunt-end ligation with T4 DNA ligase (New England Biolabs) to generate six recombinant viruses (rGI, rGIII/GI UTR, rGIII/GI C-E, rGIII/GI NS1-5, rGIII/GI NS1-3 and rGIII/GI NS4-5). We replaced the pCMV GI infectious cDNA clone with the corresponding gene fragment of GIII virus using the protocol of Gibson assembly reaction (New England Biolabs) and generated two additional recombinant viruses, rGI/GIII NS1-5 and rGI/GIII NS1-3 (Fig 5A). All fragments were amplified by PCR reactions (KOD, Novagen). PCR templates and primers are listed in S1 Table. cDNA constructs, extracted from the transformed competent cells using Mini-prep kit (Qiagen), were sequenced to authenticate the complete viral genome insert.

The site-specific mutation was individually introduced into pCMV GIII/GI NS1-3 or pCMV GIII by site-directed mutagenesis using the following reaction mixes: 1.5 mM MgSO_4_, 0.2 mM dNTPs, 0.4 mM mutagenesis primers (S1 Table), 0.5U KOD Hot Start DNA polymerase (KOD, Novagen), and the respective cDNA clones. Mutated clones were identified in cDNA constructs, extracted from the transformed competent cells using Mini-prep kit (Qiagen), and sequenced to authenticate the complete viral genome insert.

### Recovery and amplification of the recombinant JEVs (rJEVs)

BHK-21 cells were seeded into 12-well plates and grown at 37°C overnight. The next day, the mixture of 1 μg of the infectious clone and Opti-MEM (Life Technologies) was added into a mixture of Lipofectamine 2000 (Life Technologies) and Opti-MEM, and then the final mixture was incubated at room temperature for 30 minutes. Next the mixture was added onto the 80% confluent cells for five hours at 37°C, and then replaced with the culture medium. After a 3- to 4-day incubation, the production of rJEVs was detected in the transfected cells by an immunofluorescence assay utilizing mouse anti-JEV NS1 antibody (provided by Dr. Yi-Ling Lin from Academic Sinica, Taiwan). The recombinant viruses secreted from transfected cells were harvested and subsequently amplified in C6/36 cells. Virus plaques were identified by plaque assay. The viral RNA was extracted from virus-infected C6/36 cells with RNeasy mini kit (Qiagen), and transcribed into cDNA with the JEV 3’UTR primer 5’-AGATCCTGTGTTCTTCCTCA-3’ using the Superscript III transcription reaction (Thermo Fisher Scientific). The complete genome of recombinant virus was confirmed by sequencing the PCR products amplified from the viral cDNA template.

### Statistical analysis

The statistical analysis was performed by GraphPad Prism v5.01. Student’s two-tailed *t*-test was used to compare two data groups. The multiple-group comparison was calculated by One-way ANOVA, and the post test analysis performed by using Turkey’s Multiple or Dunnett’s Multiple Comparison Test. P<0.05, the significant difference in the two-group and multiple-group comparison.

## Supporting information

S1 Fig Western blot analysis of viral NS3 protein in JEV-infected cells. (A-L) The cell lysates of JEV-infected C6/36 cells at 28°C (A and D), CTR cells at 28°C (B and E) or at 34°C (C and F), VERO (G and J), PK-15 (H and K), and DF-1 (I and L) cells at 41°C were collected at 48 HPI. The viral NS3 protein and cellular α-tubulin or β-actin were detected by Western blot using mouse anti-NS3 protein, anti-α-tubulin or anti-β-actin antibodies. (D-F and J-L) The ratio of band intensity (NS3/α-tubulin) or (NS3/β-actin) was estimated by ImageJ version 1.44 with Mean and SEM calculated from triplicates. The difference in relative intensity was calculated using one-way ANOVA followed by Turkey’s Multiple Comparison Test. A significant difference is indicated by an asterisk (P< 0.05).
(TIF)

S2 FigCell viability after GIII and GI JEV infection.GIII CH1392 virus (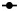), GIII T1P1 virus (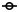), GI YL2009-4 virus (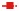), and GI TC2009-1 virus (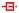) infected CTR209 (A and B), VERO (C and D), PK-15 (E and F), and DF-1 (G and H) cells at 0.5 MOI and replicated at 28°C (A), 34°C (B), 37°C (C, E, and G), and 41°C (D, F, and H). Cells only (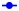) was used as control. Viable cells were count at 0, 12, 24, 36, 48, 60 HPI. Mean with SEM of the triplicates is showed. The difference of cell number was analyzed with one-way ANOVA followed by Turkey’s Multiple Comparison Test. A significant genotype-specific difference is indicated by an asterisk (P< 0.05).(TIF)

S3 FigThe growth curve of GIII and GI JEVs in chicken- and duck-derived cell lines.GIII CH1392 virus (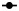), GIII T1P1 virus (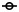), GI YL2009-4 virus (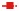), and GI TC2009-1 virus (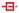) -infected CER (A and C) and DE (B and D) cells at 0.5 MOI and replicated at 37°C (A and B) and 41°C (C and D). The viral titer (ffu/ml) was determined for each supernatant at 12, 24, 36, 48, 60 hours post infection (HPI) by micro-antigen focus assay. Mean with SEM of the triplicates is shown. The difference of viral titer was analyzed with one-way ANOVA followed by Turkey’s Multiple Comparison Test. A significant genotype-specific difference is indicated by an asterisk (P< 0.05).(TIF)

S4 FigThermal stability of GIII and GI JEVs.GIII CH1392 virus (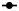), GIII T1P1 virus (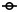), GI YL2009-4 virus (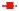), and GI TC2009-1 virus (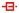) were incubated at 37°C (A) and 41°C (B), and collected at 0, 3, 6, 9, 12 hours post treatment (HPT). Infectivity of viral particles was examined by the micro-antigen focus assay and shown as 100% at 0 HPT. The infectious curve was fitted by nonlinear regression with one phase decay (A and B) and the half-life of infectious particles (C) was calculated using GraphPad Prism v5.01.(TIF)

S5 Fig Comparison of RNAemia in experimentally-infected pigs and poultry using GI and GIII JEVs. (A-C) The viral RNA in the plasma of JEV-infected pigs (A), chickens (B), and ducklings (C) was measured by real-time RT-PCR at 2 DPI. The detection limit is indicated as a dotted line. A dot plus a horizontal line represents an individual animal and a mean RNA-copy number of the group, respectively. The statistics comparing either two or four viruses were determined by a Student’s two-tailed *t*-test or one-way ANOVA followed by Turkey’s Multiple Comparison Test. The statistical difference is noted by an asterisk (P< 0.05).
(TIF)

S6 Fig Computational prediction of the secondary structure formed by 5’UTR and 3’UTR of JEV genome. The viral RNA sequence, beginning with 151 nucleotides from the 5’terminal followed by poly(A) linker and 118 nucleotides from 3’terminal, is used to predict the cyclization structure at 37°C and 41°C with mfold web server. The ΔG value of the cyclization structure is indicated.
(TIF)

S7 Fig Detection of RNAemia in rGIII and rGIII/GI NS1-3 viruses-infected pigs and chickens. (A and B) The viral RNA in plasma collected from the infected pigs (A) at days 0 to 8, and from the infected chickens (B) at 60 HPI, respectively, were measured by real-time RT-PCR. Each dot represents an individual animal. A horizontal dotted line indicates the detection limit. Mean with SEM is showed. The RNAemia in chicken plasma was statistically analyzed with one-way ANOVA, followed by Turkey’s Multiple Comparison Test. A significant difference is indicated by an asterisk ([B], P< 0.05).
(TIF)

S8 Fig The NS2B/NS3 substitutions influence the development of RNAeima in rJEV-infected chickens. (A and B) The viral RNA in collected plasma was detected at 60 HPI (A) or 48 HPI (B) and measured by real-time RT-PCR. A horizontal dot line represents an individual animal and mean of a group respectively. Error bars indicate SEM. A horizontal dot line indicates the detection limit. The statistical analysis was determined by one-way ANOVA followed by Dunnett’s Multiple Comparison Test utilizing rGIII/ GI NS1-3 virus (A) or rGIII virus (B) as a control. A significant difference is showed as a red dot (P< 0.05).
(TIF)

S9 Fig The infectivity rate of rGIII and rGI viruses in infectious center assays. rJEVs were incubated with VERO, PK-15, and DF-1 cells at an MOI of 5 at 37°C for 1 hour. Then, the infected cells were serially diluted and used to infect VERO cells using a micro-antigen focus assay. The number of infected cells releasing infectious particles was estimated and shown as ffu. The infectivity rate was calculated as the number of ffu divided by number of infected cells.
(TIF)

S10 Fig Comparison of the size of rJEVs-forming foci by micro-antigen focus assay utilizing VERO cells. (A) The focus was viewed by light microscopy. (B) The size of focus was estimated by ImageJ version 1.44. Mean with SEM for four foci is showed. The difference in size was calculated using one-way ANOVA followed by Turkey’s Multiple Comparison Test.
(TIF)

S1 Table Primers used to construct recombinant and site-direct mutated infectious clones. (PDF)

S2 Table Specific NS2B/NS3 substitutions among five JEV genotypes. (DOCX)

S1 Methods SDS PAGE, Western blot, and Real-time RT-PCR. (DOCX)
