# Supplementary figures and images for: NS2B/NS3 mutations enhance the infectivity of genotype I Japanese encephalitis virus in amplifying hosts
Source: PLoS Pathog. 2019 Aug 5;15(8):e1007992. doi: 10.1371/journal.ppat.1007992 (PMC6695206; doi:10.1371/journal.ppat.1007992)

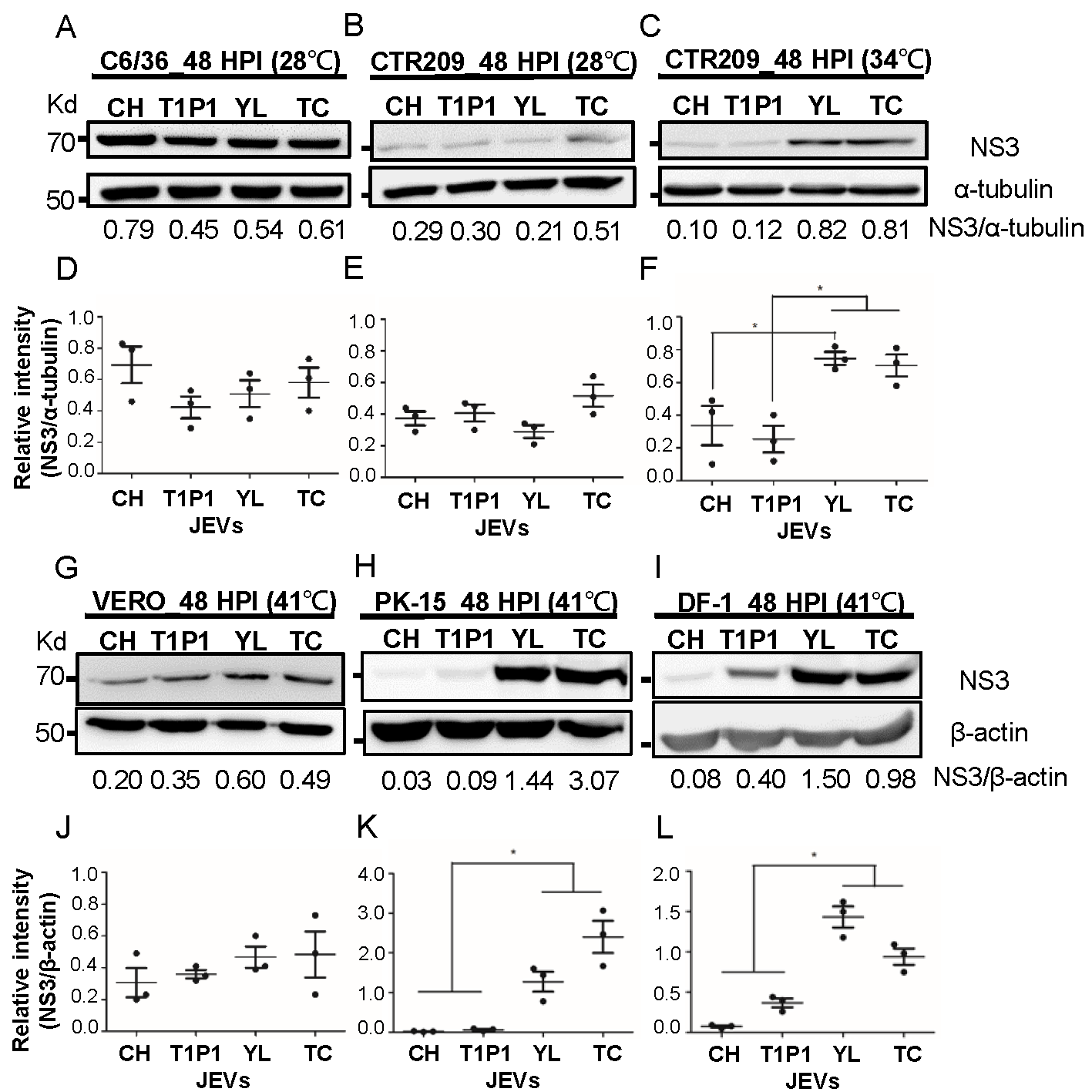

Supplement: S1 Fig — (A-L) The cell lysates of JEV-infected C6/36 cells at 28°C (A and D), CTR cells at 28°C (B and E) or at 34°C (C and F), VERO (G and J), PK-15 (H and K), and DF-1 (I and L) cells at 41°C were collected at 48 HPI. The viral NS3 protein and cellular α-tubulin or β-actin were detected by Western blot using mouse anti-NS3 protein, anti-α-tubulin or anti-β-actin antibodies. (D-F and J-L) The ratio of band intensity (NS3/α-tubulin) or (NS3/β-actin) was estimated by ImageJ version 1.44 with Mean and SEM calculated from triplicates. The difference in relative intensity was calculated using one-way ANOVA followed by Turkey’s Multiple Comparison Test. A significant difference is indicated by an asterisk (P< 0.05). (TIF) [file ppat.1007992.s001.tif]

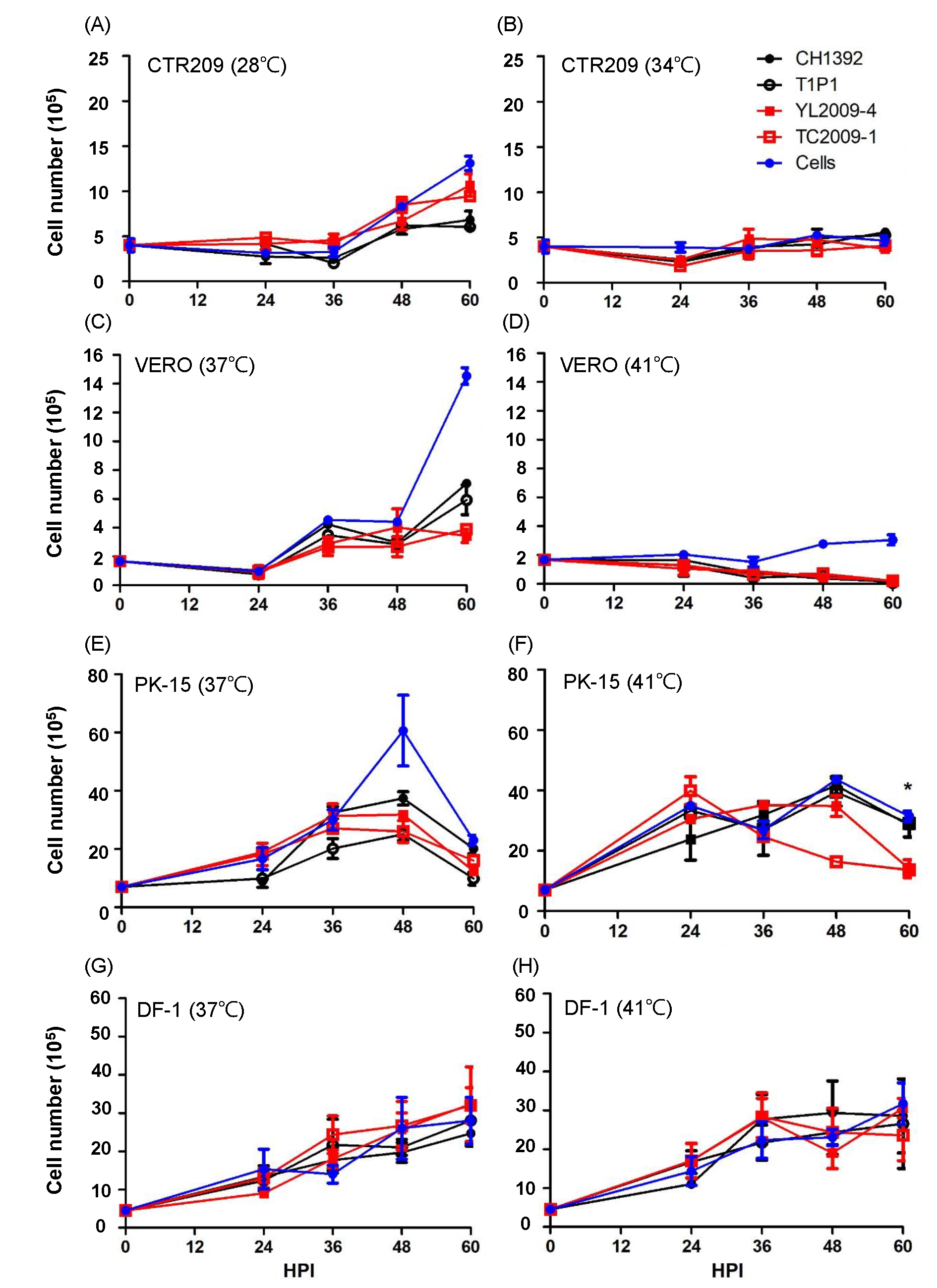

Supplement: S2 Fig — GIII CH1392 virus (), GIII T1P1 virus (), GI YL2009-4 virus (), and GI TC2009-1 virus () infected CTR209 (A and B), VERO (C and D), PK-15 (E and F), and DF-1 (G and H) cells at 0.5 MOI and replicated at 28°C (A), 34°C (B), 37°C (C, E, and G), and 41°C (D, F, and H). Cells only () was used as control. Viable cells were count at 0, 12, 24, 36, 48, 60 HPI. Mean with SEM of the triplicates is showed. The difference of cell number was analyzed with one-way ANOVA followed by Turkey’s Multiple Comparison Test. A significant genotype-specific difference is indicated by an asterisk (P< 0.05). (TIF) [file ppat.1007992.s002.tif]

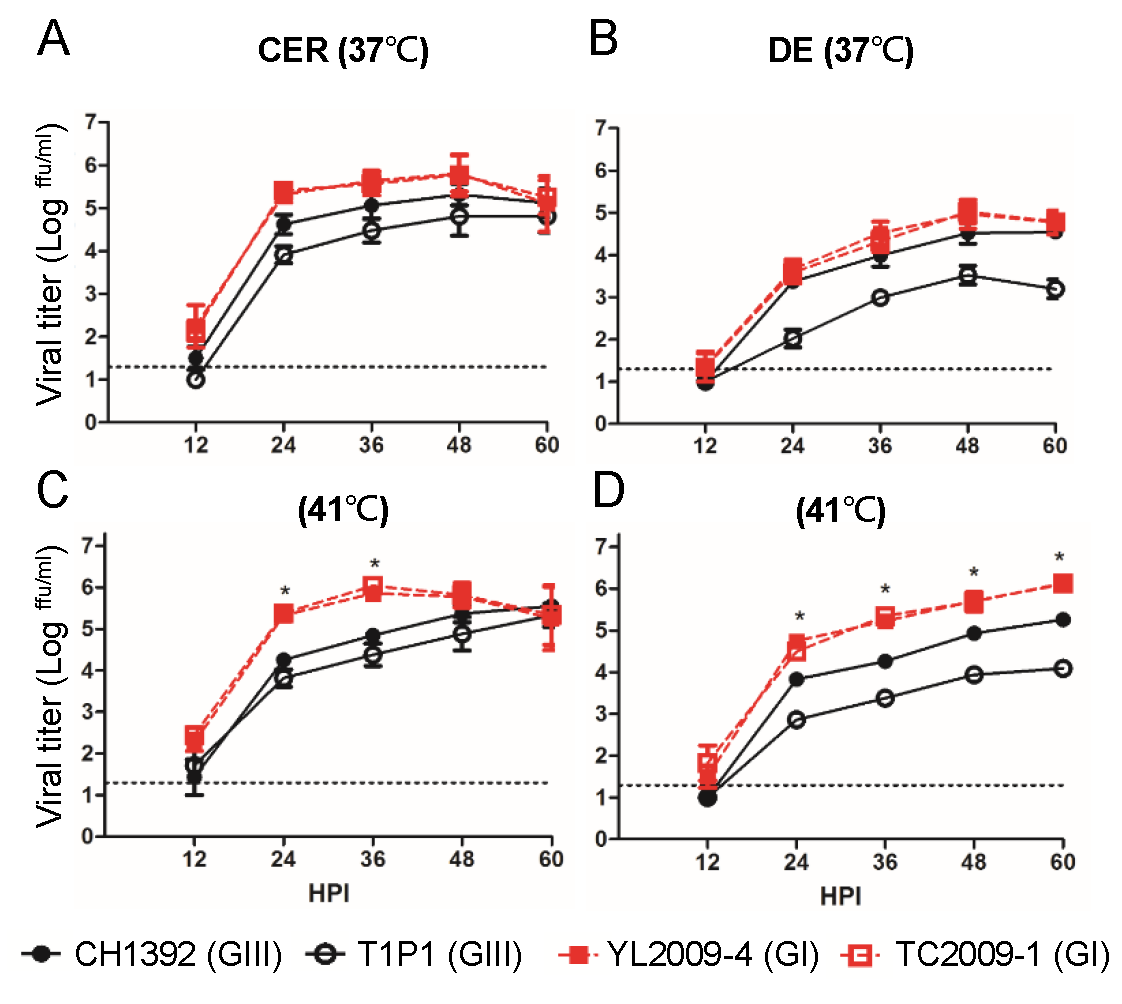

Supplement: S3 Fig — GIII CH1392 virus (), GIII T1P1 virus (), GI YL2009-4 virus (), and GI TC2009-1 virus () -infected CER (A and C) and DE (B and D) cells at 0.5 MOI and replicated at 37°C (A and B) and 41°C (C and D). The viral titer (ffu/ml) was determined for each supernatant at 12, 24, 36, 48, 60 hours post infection (HPI) by micro-antigen focus assay. Mean with SEM of the triplicates is shown. The difference of viral titer was analyzed with one-way ANOVA followed by Turkey’s Multiple Comparison Test. A significant genotype-specific difference is indicated by an asterisk (P< 0.05). (TIF) [file ppat.1007992.s003.tif]

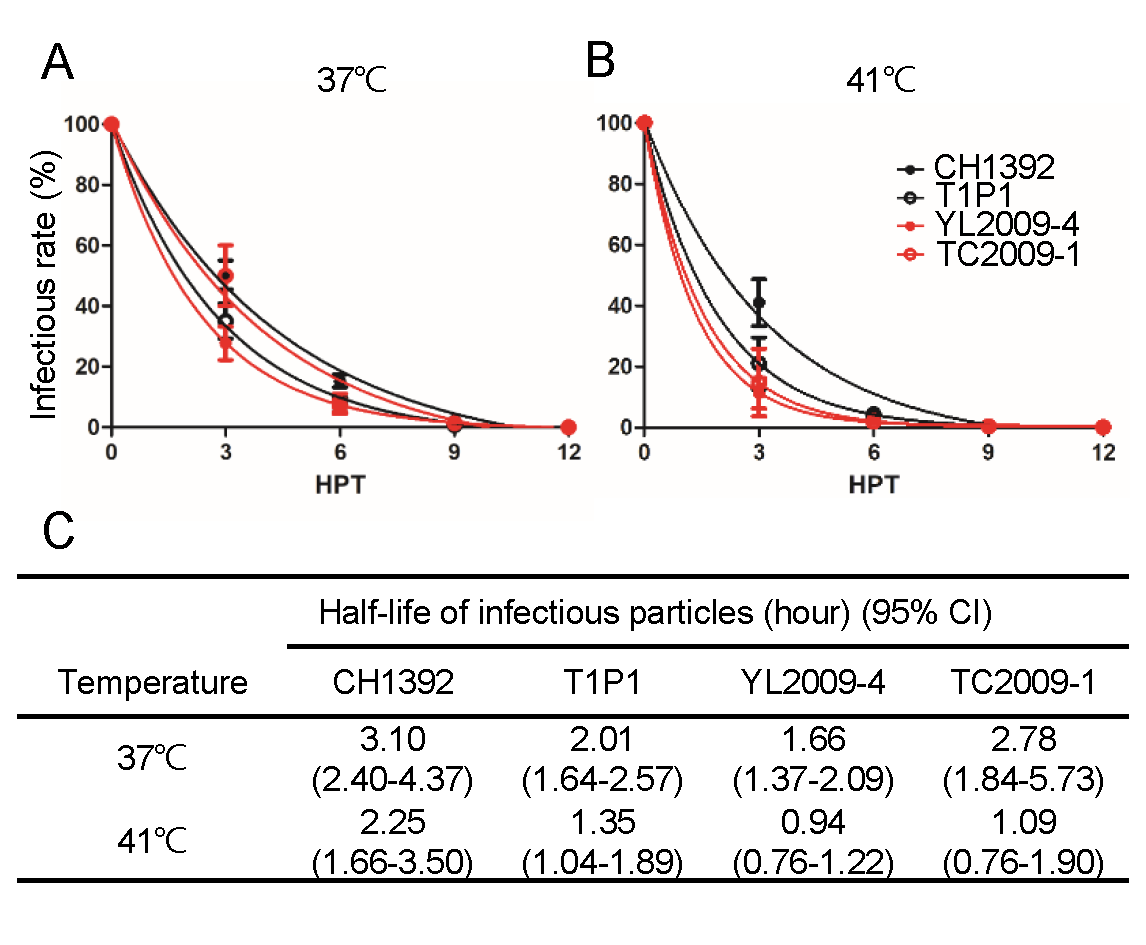

Supplement: S4 Fig — GIII CH1392 virus (), GIII T1P1 virus (), GI YL2009-4 virus (), and GI TC2009-1 virus () were incubated at 37°C (A) and 41°C (B), and collected at 0, 3, 6, 9, 12 hours post treatment (HPT). Infectivity of viral particles was examined by the micro-antigen focus assay and shown as 100% at 0 HPT. The infectious curve was fitted by nonlinear regression with one phase decay (A and B) and the half-life of infectious particles (C) was calculated using GraphPad Prism v5.01. (TIF) [file ppat.1007992.s004.tif]

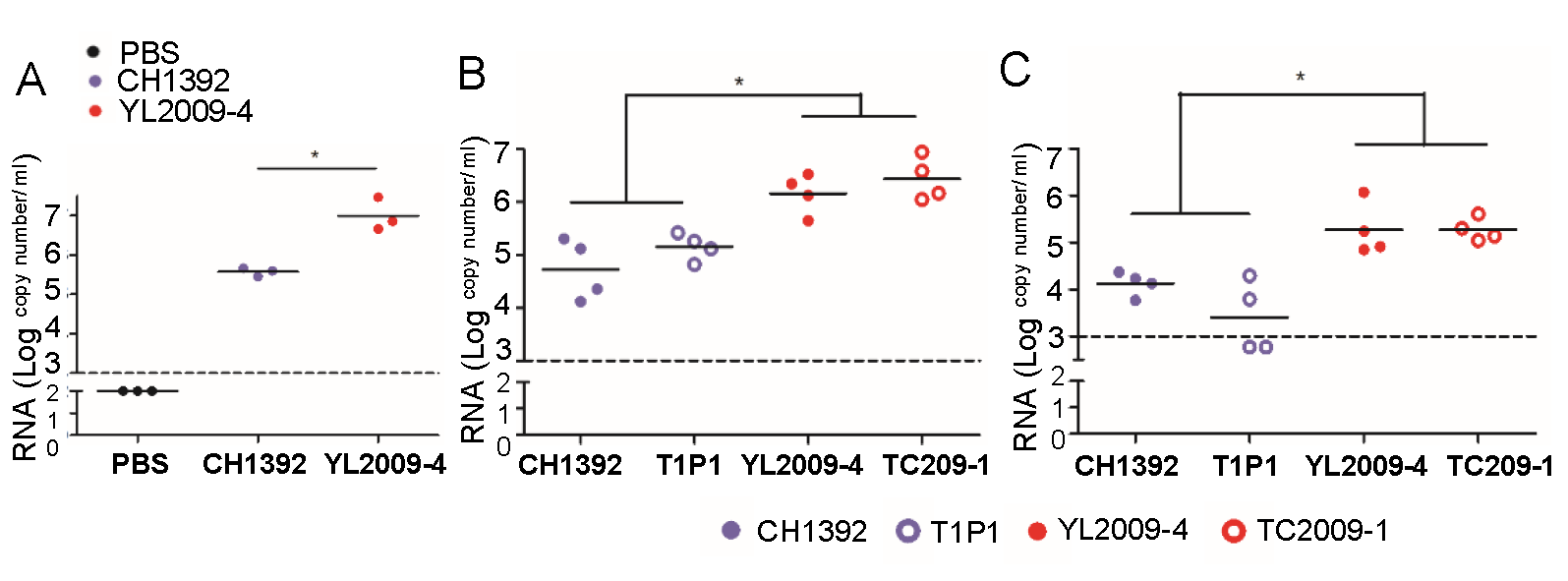

Supplement: S5 Fig — (A-C) The viral RNA in the plasma of JEV-infected pigs (A), chickens (B), and ducklings (C) was measured by real-time RT-PCR at 2 DPI. The detection limit is indicated as a dotted line. A dot plus a horizontal line represents an individual animal and a mean RNA-copy number of the group, respectively. The statistics comparing either two or four viruses were determined by a Student’s two-tailed t-test or one-way ANOVA followed by Turkey’s Multiple Comparison Test. The statistical difference is noted by an asterisk (P< 0.05). (TIF) [file ppat.1007992.s005.tif]

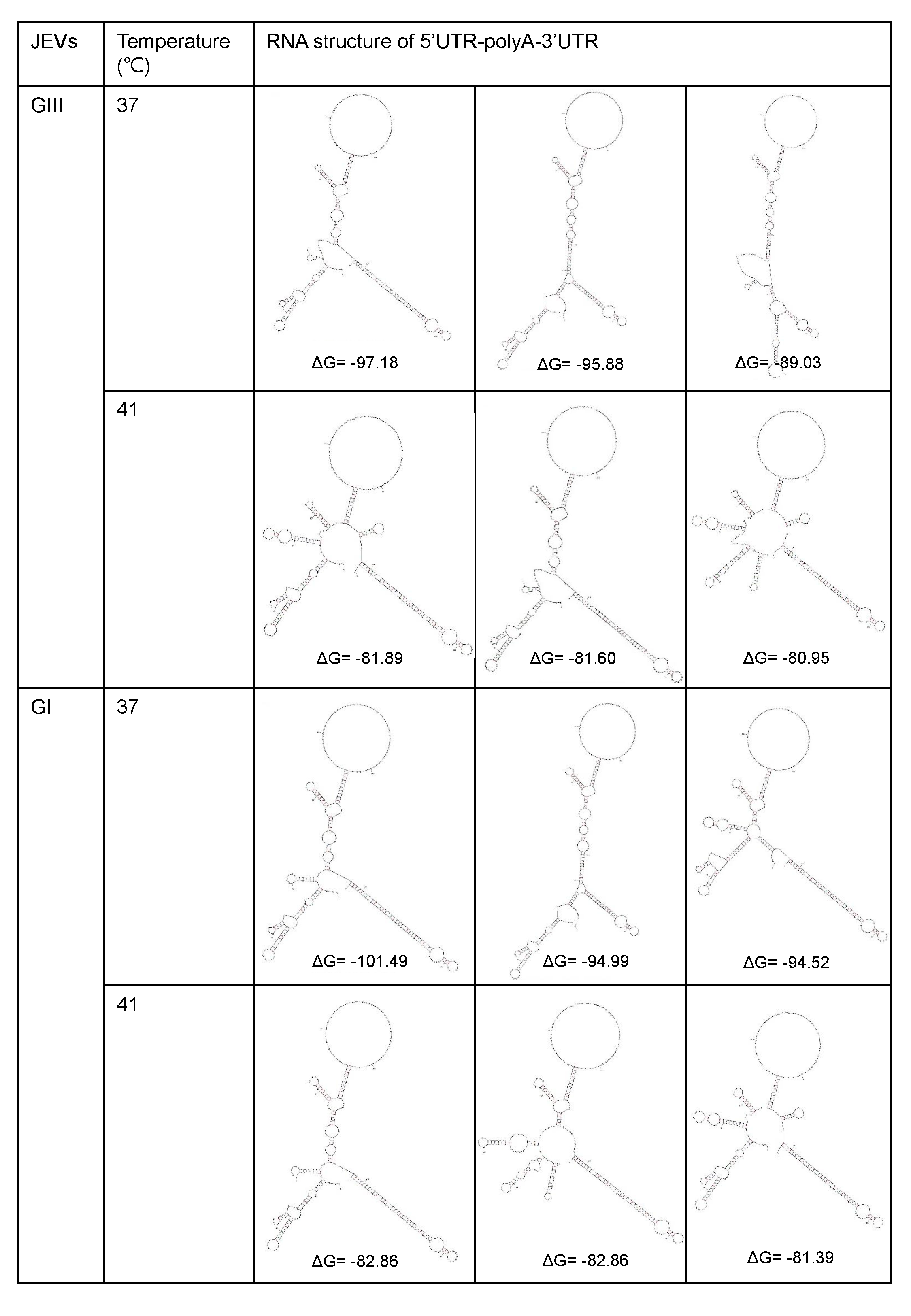

Supplement: S6 Fig — The viral RNA sequence, beginning with 151 nucleotides from the 5’terminal followed by poly(A) linker and 118 nucleotides from 3’terminal, is used to predict the cyclization structure at 37°C and 41°C with mfold web server. The ΔG value of the cyclization structure is indicated. (TIF) [file ppat.1007992.s006.tif]

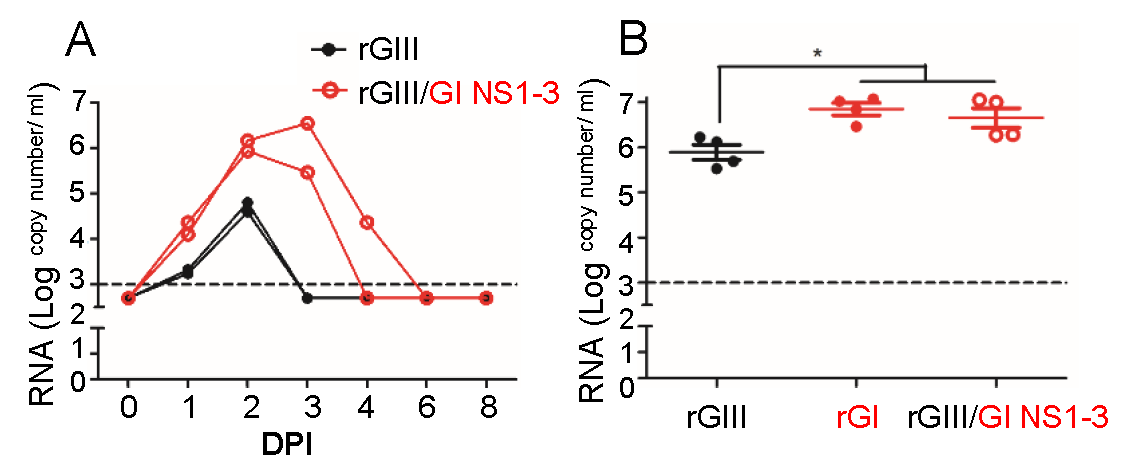

Supplement: S7 Fig — (A and B) The viral RNA in plasma collected from the infected pigs (A) at days 0 to 8, and from the infected chickens (B) at 60 HPI, respectively, were measured by real-time RT-PCR. Each dot represents an individual animal. A horizontal dotted line indicates the detection limit. Mean with SEM is showed. The RNAemia in chicken plasma was statistically analyzed with one-way ANOVA, followed by Turkey’s Multiple Comparison Test. A significant difference is indicated by an asterisk ([B], P< 0.05). (TIF) [file ppat.1007992.s007.tif]

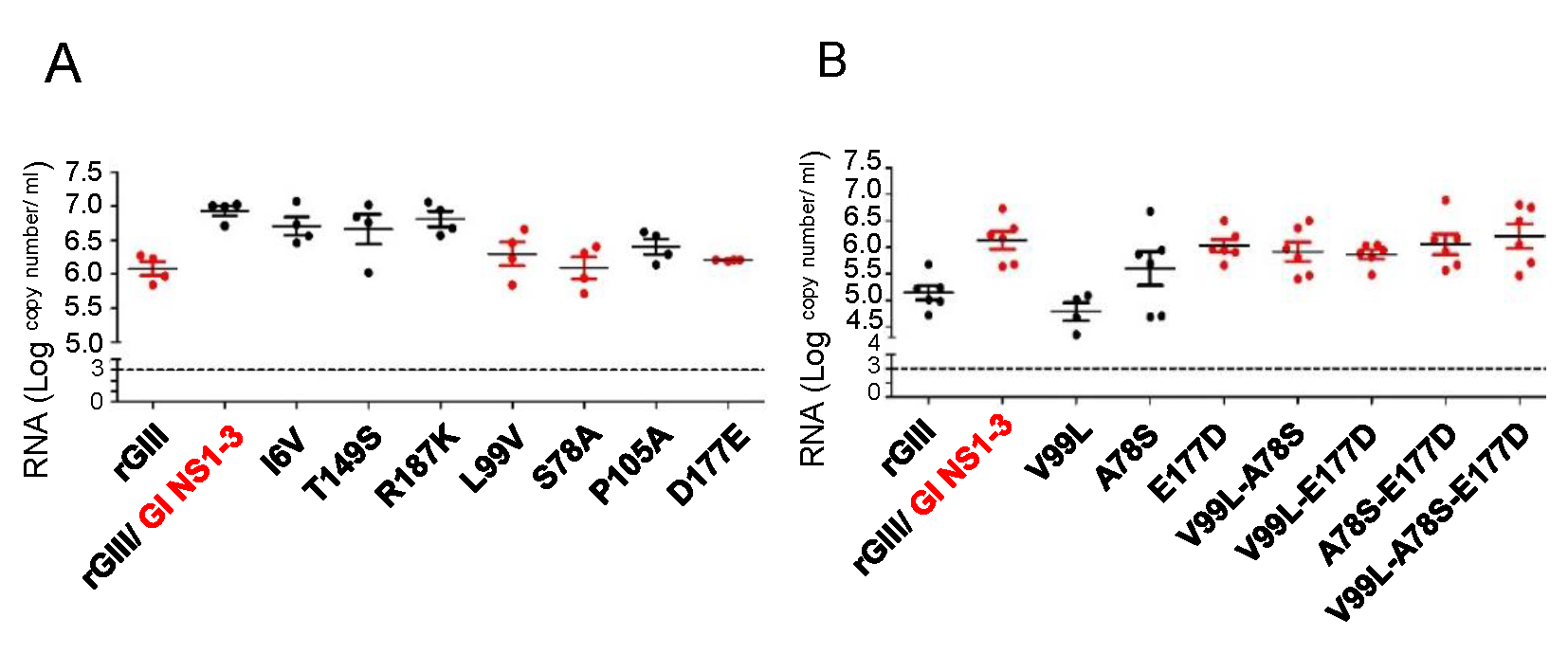

Supplement: S8 Fig — (A and B) The viral RNA in collected plasma was detected at 60 HPI (A) or 48 HPI (B) and measured by real-time RT-PCR. A horizontal dot line represents an individual animal and mean of a group respectively. Error bars indicate SEM. A horizontal dot line indicates the detection limit. The statistical analysis was determined by one-way ANOVA followed by Dunnett’s Multiple Comparison Test utilizing rGIII/ GI NS1-3 virus (A) or rGIII virus (B) as a control. A significant difference is showed as a red dot (P< 0.05). (TIF) [file ppat.1007992.s008.tif]

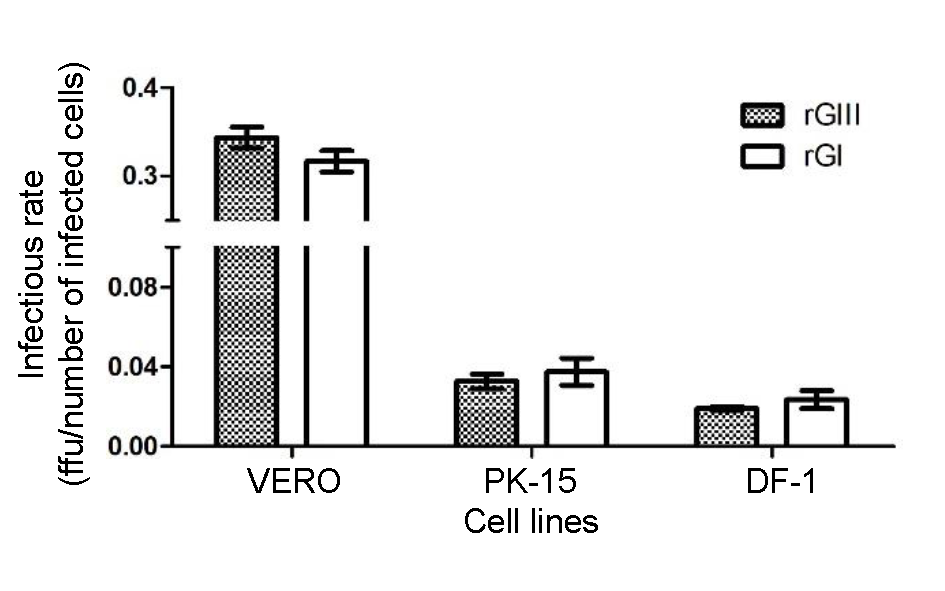

Supplement: S9 Fig — rJEVs were incubated with VERO, PK-15, and DF-1 cells at an MOI of 5 at 37°C for 1 hour. Then, the infected cells were serially diluted and used to infect VERO cells using a micro-antigen focus assay. The number of infected cells releasing infectious particles was estimated and shown as ffu. The infectivity rate was calculated as the number of ffu divided by number of infected cells. (TIF) [file ppat.1007992.s009.tif]

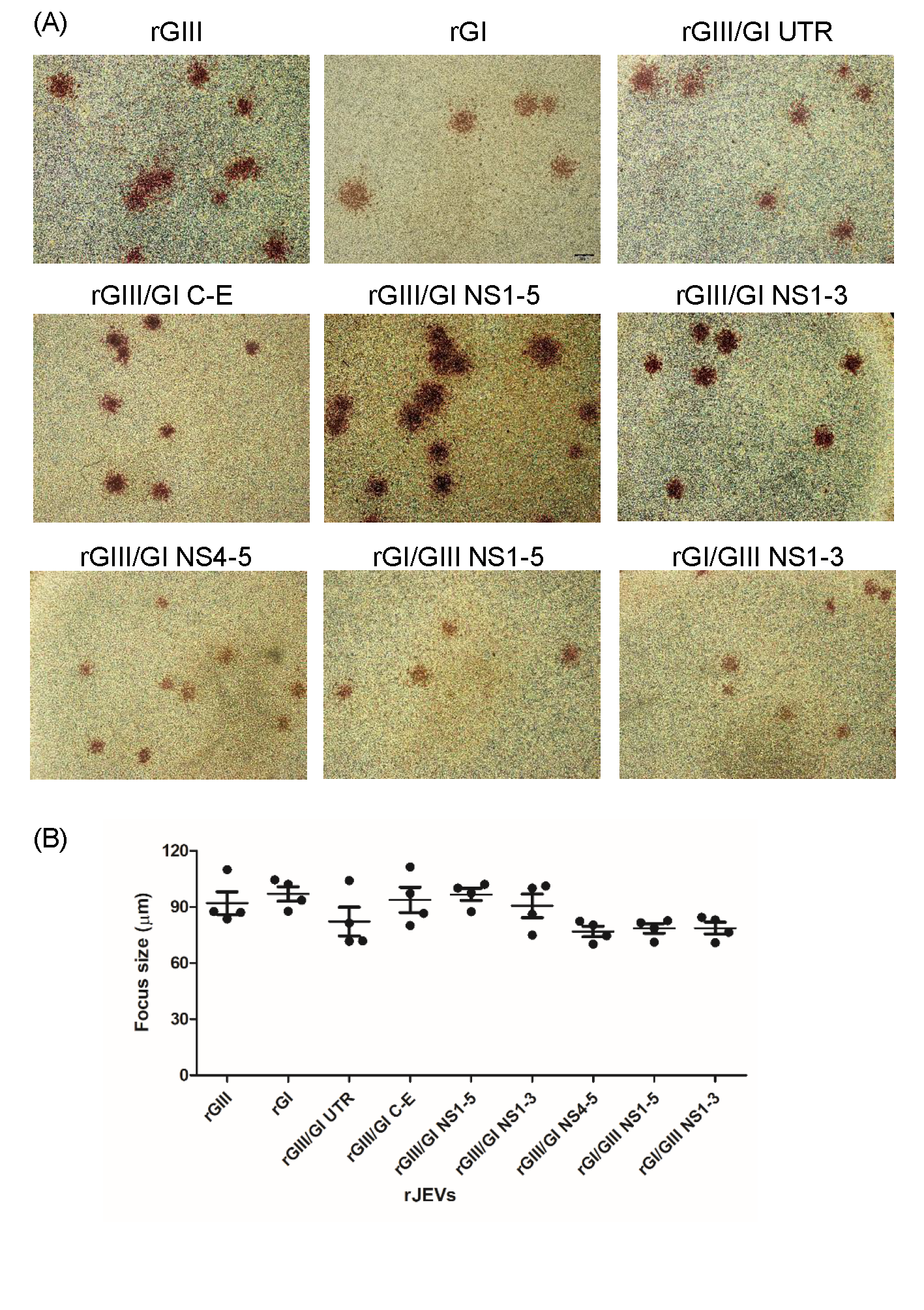

Supplement: S10 Fig — (A) The focus was viewed by light microscopy. (B) The size of focus was estimated by ImageJ version 1.44. Mean with SEM for four foci is showed. The difference in size was calculated using one-way ANOVA followed by Turkey’s Multiple Comparison Test. (TIF) [file ppat.1007992.s010.tif]
