# Supplementary material for: NS2B/NS3 mutations enhance the infectivity of genotype I Japanese encephalitis virus in amplifying hosts
Source: PLoS Pathog. 2019 Aug 5;15(8):e1007992. doi: 10.1371/journal.ppat.1007992 (PMC6695206; doi:10.1371/journal.ppat.1007992)
Supplement: S1 Table — (PDF) [file ppat.1007992.s011.pdf]

S1 Table. Primers used to construct recombinant and site-direct mutated infectious clones.

Infectious clones	Primer name	Primers 5'-3'	Templates
pCMV GI	HDVr-F	GGGTCGGCATGGCATCTC	pCMV GIII
	CMV-R	GGTTCACTAAACCAGCTCTGCTTATATAG	
	JGI 5'UTR-F	5'p-AGAAGTTTATCTGTGTGAACTTCTTGGC	GI virus cDNA
	JGI 3'UTR-R	5'p-AGATCCTGTGTTCTTCCTCACCAC	
pCMV GIII/GI UTR	JGI 3'UTR-F	TAGATAGGATTAAGTCATGTGTGTAATGTG	pCMV GI
	JGI 5'UTR-R	GGTTGTTCTTCCGTTCTAAAAAAC	
	Vector-GIII capsid-F	tttagaacggaagaacaaccATGACTAAAAAACCAGGAGG	pCMV GIII
	Vector-GIII NS5-R	acatgacttaatcctatctaGATGACCCTGTCTTCCTG	
pCMV GIII/GI C-E	JGIII NS1-F	GACACTGGATGTGCCATTGAC	pCMV GIII
	JGIII 5'UTR-R	GGTTATCTTCCGTTCTAAAAAACTGTT	
	JGI capsid-F	5'p-ATGACTAAAAAACCAGGAGGGCC	GI virus cDNA
	JGI E-R	5'p-GGCATGCACGTTGGTCGCTAA	
pCMV GIII/GI NS1-5	JGIII 3'UTR-F	TAGTGTGATTTAAGGTAGAAAAGTAGACTATGT	pCMV GIII
	JGIII E-R	AGCATGCACATTGGTCGCTA	
	JGI NS1-F	5'p-GATACTGGCTGTGCCATTGATATCAC	GI virus cDNA
	JGI NS5-R	5'p-AATGACCCTGTCCTCCTGAATCAATAC	
pCMV GIII/GI NS1-3	JGIII 3'UTR-F	TAGTGTGATTTAAGGTAGAAAAGTAGACTATGT	pCMV GIII/GI NS1-5
	JGI NS3-R	TCTCTTGCCCGCTGCAAAATCC	
	vector-GIII NS4A-F	attttgcagcgggcaagagaTCAGCCGTTAGCTTCATAG	pCMV GIII

	vector-GIII NS5-R	ttctaccttaaatcacactaGATGACCCTGTCTTCCTG	
pCMV GIII/GI NS4-5	JGIII 3'UTR-F	TAGTGTGATTTAAGGTAGAAAAGTAGACTATGT	pCMV GIII
	JGIII NS3-R	TCTCTTTCCTGCTGCGAAGTCT	
	vector-GI NS4A-F	acttcgcagcaggaaagagaTCAGCCGTCAGTTTCATAG	pCMV GI
	vector-GI NS5-R	tacttttctaccttaaatcacactaAATGACCCTGTCCTCCTGAATCAATACATC	
pCMV GI/GIII NS1-5	JGI 3'UTR-F	TAGATAGGATTAAGTCATGTGTGTAATGTG	pCMV GI
	JGI E-R	5'p-GGCATGCACGTTGGTCGCTAA	
	Vector-GIII NS1-F	tagcgaccaacgtgcatgccGACACTGGATGTGCCATTG	pCMV GIII
	Vector-GIII NS5-R	acatgacttaatcctatctaGATGACCCTGTCTTCCTG	
pCMV GI/GIII NS1-3	JGI NS4A-F	TCAGCCGTCAGTTTCATAGAGGTG	pCMV GI
	JGI E-R	5'p-GGCATGCACGTTGGTCGCTAA	
	vector-GIII NS1-F	tagcgaccaatgtgcatgctGACACTGGATGTGCCATTG	pCMV GIII
	vector-GIII NS3-R	tctatgaaactgacggctgaTCTCTTTCCTGCTGCGAAG	
pCMV GIII/GI NS1-3 mutants	NS1 Q51K	ACCCCGCACACTCCTTCcttATGTGCTTTATGGACGATCTTC	pCMV GIII/GI NS1-3
	NS1 S70A	CATTCAGTTCGTCCCGCACggcTTCCCACATCTGGTGTTCCA	
	NS1 R147H	GCATGCTATTCCAGGCTCTgtgCTCATCAGGGCATTCCTTTG	
	NS1 L206Y	CTCAAGTTTCCAGGTGTCATTgtaACGGCTCTCAATCCAGTATGA	
	NS1 R251K	TCTCCGGTTGTGCTTGCTtttCGGTCCAGCTATGGTGTGT	
	NS1 I298V	CTTTCCACTGTCAGTAGTAGTTCTgacTGAGGGACCCCTCTTGC	
	NS2A I6V	CCCAGCTGAAAAGGGTCaacCATTTCGCCGTTGAAAGC	
	NS2A A97T	CTCTTGGTTCGTCCATCTcgtGCTAAGCATGTTCATGACCAGA	
	NS2A T149S	GACTGGCATGGCAACggaAGAAGTTGTGGGAAATGTGATCG	

	NS2A R187K	TCTTCTTCGCCATGGTCTTtttCCTCTCTTGCAGCAGGGA	
	NS2B E65D	GGCTACTTCCTGTGATTGCAGCatcCATCTCCCAGCTGATATCGG	
	NS2B L99V	GCCGATACAAGACATACGCAAgacCCAGACTTTCCATGGAACACC	
	NS3 S78A	CTCCACGGGCCTCCATAagcTATGCGGTCTTCCTTCACACT	
	NS3 P105A	GGTTTTGTCTGGATGTTTACTGCagcTTTCCCTGGTTCCACCACG	
	NS3 D177E	AGCATGCTTGGAGTGTAGGCttcTGGAACTGGTTCCTCTTGACG	
	NS3 S182N	AGTCATCTGTCTCTTTTTCAGCATgttTGGAGTGTAGGCATCTGGAAC	
pCMV GIII NS2B/NS3 mutants	NS2B V99L	AATGCAAGACATGCGCAGgagCCAGACCTTCCATGGAACAC	pCMV GIII
	NS3 A78S	CTCCATGGGCCTCCGTAgctTATGCGGTCTTCTTTCACACTAC	
	NS3 E177D	CAACATGTTTGGGGTGTAAGCatcTGGGACTGGTTCCTCCTG	
