# Supplementary material for: NS2B/NS3 mutations enhance the infectivity of genotype I Japanese encephalitis virus in amplifying hosts
Source: PLoS Pathog. 2019 Aug 5;15(8):e1007992. doi: 10.1371/journal.ppat.1007992 (PMC6695206; doi:10.1371/journal.ppat.1007992)
Supplement: S2 Table — (DOCX) [file ppat.1007992.s012.docx]

S2 Table. Specific NS2B/NS3 substitutions among five JEV genotypes

| JEV genotype^a^ | NS2B | | NS3 | | | |
| --- | --- | --- | --- | --- | --- | --- |
|  | 65 | 99 | 78 | 105 | 177 | 182 |
| GI | E | L | S | P | D | S |
| GII | D | L | S | A | E | S |
| GIII | D | V | A | A | E | N |
| GIV | D | V | A | A | E | S |
| GV | D | V | T | A | D | N |

^a^GI and GIII JEVs used were the same in Table 1. Four GII JEVs, one GIV JEV, and two GV JEVs NS2B/NS3 sequences were available and showed here. GII FU (AAF73859), GII JKT654 (ADT63077), GII WTP-70-22 (ADT63076), and GII Bennett (ADT63075) strains; GIV JKT6468 (AAP39942) strain; GV Tengah (AJE59927) and Muar (ADX31663) strains.
