# Supplementary material for: NS2B/NS3 mutations enhance the infectivity of genotype I Japanese encephalitis virus in amplifying hosts
Source: PLoS Pathog. 2019 Aug 5;15(8):e1007992. doi: 10.1371/journal.ppat.1007992 (PMC6695206; doi:10.1371/journal.ppat.1007992)
Supplement: S1 Methods — (DOCX) [file ppat.1007992.s013.docx]

**Supporting Information Supplemental methods**

**SDS PAGE and Western blot**

Virus-infected cells were lysed with RIPA lysis buffer (BioVision) with the addition of RNase inhibitor (Roche) and agitated at 4°C for 30 minutes. The sample including viral and cellular proteins was recovered from the supernatant of the lysed cells after centrifugation. We mixed the sample with 5X reducing sample buffer (315 mM Tris, pH 6.8, 50% glycerol, 5% SDS. 0.025% bromophenol blue, 12% 2-ME), and denatured it in boiling water for 10 minutes. Then, the denatured proteins were loaded onto a 10% SDS gel and separated by the electrophoresis. The separated proteins were transferred from gel to nitrocellulose membrane, blocked by 5% skim milk, and detected with mouse anti-α tubulin monoclonal antibody (MAb) (Novus Biologicals), anti-β actin MAb (Novus Biologicals), and mouse anti-flavivirus NS3 protein MAb (Yao-Hong Biotechnology) followed by HRP-conjugated goat anti-mouse IgG (H+L) (Jackson ImmunoReserch, West Grove, PA). The predicted bands were developed by use of the LumiGOLD ECL Western Blot Detection Kit (SignaGen Laboratories, Gaithersburg, MD) and quantified by use of the ImageJ version 1.44 (NIH, Bethesda, MD).

**Real-time RT-PCR**

10^6^ VERO cells were added to the plasma collected from animals. Viral and cellular RNA were extracted from this mixture with RNeasy mini kit (Qiagen), and transcribed into cDNA with random primers in the Superscript III transcription reaction (Thermo Fisher Scientific). The real-time RT-PCR reaction was assembled using IQTM SYBR® Green Supermix (Bio-Rad), cDNA transcripts, and JEV 3’UTR primers (5’-TGGGTTAMCAAAGCCGTTGA-3’ and 5’-ACATACTTCGGCGCTCTGTG-3’) or actin primers (5’-TCCTGTGGCATCCACGAAACT-3’ and 5’-GAAGCATTTGCGGTGGACGAT-3’) and performed with CFX connectTM Real-Time PCR Detection System (Bio-Rad). Viral RNA quantity was calculated using a full-length cDNA plasmid as the copy-number standard, normalized with actin mRNA by using the Bio-Rad CFX Manager v3.1 (Bio-Rad), and expressed as the copy number per milliliter.
